# Spatial regulation of monolignol biosynthesis and laccase genes control developmental and stress-related lignin in flax

**DOI:** 10.1186/s12870-017-1072-9

**Published:** 2017-07-14

**Authors:** Julien Le Roy, Anne-Sophie Blervacq, Anne Créach, Brigitte Huss, Simon Hawkins, Godfrey Neutelings

**Affiliations:** University of Lille, CNRS, UMR 8576 - UGSF - Unité de Glycobiologie Structurale et Fonctionnelle, F-59000 Lille, France

**Keywords:** Flax, Laccase, Lignin, microRNA, Monolignol, Phenylpropanoids, Stress

## Abstract

**Background:**

Bast fibres are characterized by very thick secondary cell walls containing high amounts of cellulose and low lignin contents in contrast to the heavily lignified cell walls typically found in the xylem tissues. To improve the quality of the fiber-based products in the future, a thorough understanding of the main cell wall polymer biosynthetic pathways is required. In this study we have carried out a characterization of the genes involved in lignin biosynthesis in flax along with some of their regulation mechanisms.

**Results:**

We have first identified the members of the phenylpropanoid gene families through a combination of in silico approaches. The more specific lignin genes were further characterized by high throughput transcriptomic approaches in different organs and physiological conditions and their cell/tissue expression was localized in the stems, roots and leaves. Laccases play an important role in the polymerization of monolignols. This multigenic family was determined and a miRNA was identified to play a role in the posttranscriptional regulation by cleaving the transcripts of some specific genes shown to be expressed in lignified tissues. In situ hybridization also showed that the miRNA precursor was expressed in the young xylem cells located near the vascular cambium. The results obtained in this work also allowed us to determine that most of the genes involved in lignin biosynthesis are included in a unique co-expression cluster and that MYB transcription factors are potentially good candidates for regulating these genes.

**Conclusions:**

Target engineering of cell walls to improve plant product quality requires good knowledge of the genes responsible for the production of the main polymers. For bast fiber plants such as flax, it is important to target the correct genes from the beginning since the difficulty to produce transgenic material does not make possible to test a large number of genes. Our work determined which of these genes could be potentially modified and showed that it was possible to target different regulatory pathways to modify lignification.

**Electronic supplementary material:**

The online version of this article (doi:10.1186/s12870-017-1072-9) contains supplementary material, which is available to authorized users.

## Background

**Fig. 1:**
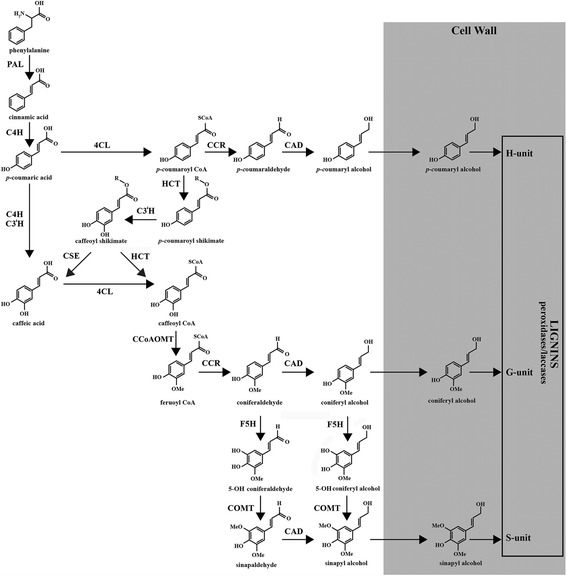
The monolignol and lignin biosynthetic pathway. 4CL: 4-coumarate:CoA ligase; BGLU: beta glucosidase; C3’H: *p*-coumarate 3-hydroxylase; C4H: cinnamate 4-hydroxylase; CAD: cinnamyl alcohol dehydrogenase; CCoAOMT: caffeoyl CoA 3-*O*-methyltransferase; CCR: cinnamoyl CoA reductase; COMT: caffeate/5-hydroxyferulate *O*-methyl-transferase; CSE: caffeoyl shikimate esterase; F5H: ferulate 5-hydroxylase; HCT: hydroxycinnamoyl-CoA:shikimate hydroxycinnamoyl transferase; PAL: phenylalanine ammonia-lyase; UGT: UDP glycosyltransferase

Lignin is among the most abundant biological polymers on earth. It is a complex aromatic molecule synthesized during the onset of the secondary cell wall (SCW) formation in plants providing stiffness proprieties for mechanical strength, hydrophobicity for water transport and contributes to defense against pests and pathogens. Lignin is produced by a complex biosynthetic pathway (Fig. 1) which also leads to the production of a wide range of phenylpropanoids with diverse and sometimes unknown functions such as hydroxycinnamic acids, flavonoids, coumarins, chalcones, phenylpropenes and stilbenes [1]. In brief, phenylalanine derived from the shikimate pathway is used as an initial substrate [2]. This amino acid is first deaminated by phenylalanine ammonia-lyase (PAL; EC 4.3.1.5), hydroxylated by cinammate 4-hydroxylase (C4H; EC 1.14.13.11) and then esterified with CoA by 4-coumarate:CoA ligase (4CL; EC 6.2.1.12) forming *p-*coumaroyl CoA. This metabolite can lead to the formation of *p*-coumaryl alcohol by the action of cinnamoyl CoA reductase (CCR; EC 1.2.1.44) and cinnamyl alcohol dehydrogenase (CAD; EC 1.1.1.195). *p-*coumaroyl CoA can also be successively transformed by hydroxycinnamoyl-CoA:shikimate hydroxycinnamoyl transferase (HCT; EC 2.3.1.133) and *p*-coumarate 3-hydroxylase (C3’H; EC 1.14.14.1) to form caffeoyl CoA that can be methoxylated by caffeoyl CoA 3-*O*-methyltransferase (CCoAOMT; EC 2.1.1.104) into feruloyl CoA. This CoA ester can be reduced into coniferaldehyde by CCR and further transformed into coniferyl alcohol by CAD. Both molecules are hydroxylated by ferulate 5-hydroxylase (F5H; EC 1.14.13) and methoxylated by caffeate/5-hydroxyferulate *O*-methyl-transferase (COMT; EC 2.1.1.68) forming sinapaldehyde and sinapyl alcohols respectively. In addition to *p*-coumaryl alcohol, both alcohols are named monolignols. These can be transported to the apoplast and transformed into radicals by laccases and peroxidases [3, 4]. The oxidative coupling of these activated monolignols leads to the production of *p*-hydroxyphenyl (H), syringyl (S) and guaiacyl (G) units respectively, present in different proportions within the lignin fractions depending on the taxonomic families, species, tissues or environment [5]. The lignin biosynthesis pathway has been comprehensively characterized in model species such as *Arabidopsis* and the identified gene models then used to search for orthologous sequences in more economically-important plants including food crops as well as woody or fiber species.

Bast fibers are present in bundles within the stem cortex between the epidermis and the xylem core in some non-woody plants. These bundles may contain only primary fibers derived from the procambium in species such as flax (*Linum usitatissimum*) or ramie (*Boehmeria nivea*) while others such as jute (*Corchorus species*), kenaf (*Hibiscus cannabinus*) or hemp (*Cannabis sativa*) contain additional secondary fibers derived from the vascular cambium. Fiber species are considered as interesting biological models because their stems contain two main populations of cells showing highly contrasted SCW compositions [6]. The xylem cells from the inner-stem tissues have a typical SCW structure with up to 30% of the cell wall weight represented by lignins whereas the bast fibers possess cellulose-rich thick SCWs with lignin contents ranging from less than 1% (ramie) to 19% (kenaf) [7]. The cell wall structure and composition is very important in these species because they determine the properties of the extracted raw materials. Flax, for instance, has been used for several thousands of years to make ropes and linen tissues and more recently, for the production of environmentally friendly fiber-based composite materials [8, 9]. To optimize the use of these materials in the future, targeted engineering of the cell wall to improve product quality will require a thorough understanding of the biosynthetic pathways leading to the production of the main cell wall polymers. Over the past years, significant efforts including genome sequencing [10], development of molecular tools [11–13] and databases [14, 15] have allowed us not only to improve our understanding of flax fiber cell wall biology, but also to identify the different gene models potentially associated with the biosynthesis of different cell wall polymers. Nevertheless, many cell wall genes are part of multigenic families and a major challenge to engineering is the identification of the individual family members involved in lignification. For non-model plants such as flax, it is important to target the correct genes from the beginning since rapid and easy transformation to obtain high amounts of transgenic material is particularly difficult [16].

To clearly identify the genes that are indeed involved in flax lignin production, we first conducted a high throughput reverse-transcriptase quantitative PCR (HT-RT-qPCR) approach to establish the expression profile of in silico-identified genes using organs/tissues with contrasted lignin contents. To further characterize these genes and because it was not possible to use large-scale reporter gene transformation, we used in situ hybridization to confirm the expression of the identified genes in lignified tissues. This type of approach has already been used to detect some lignin gene transcripts in woody plants [17–19] but never on such a large scale. We also benefited from the development of this technique in flax to show that a microRNA, *miR397*, already shown to be involved in the cleavage of several laccase transcripts [20, 21] is co-expressed in the same cells as the lignin biosynthetic genes confirming the role of miRNAs on laccase regulation in flax. In the context of a possible impact of global climate changes on the quality of plant products, and because lignin is an important component in stress responses [5], the expression of the identified genes was also analysed under a range of different environmental conditions. The high amount of data obtained by HT-RT-qPCR has enabled us to show that the expression of most of these genes is coordinated, with a probable implication of MYB transcription factors.

## Results

### Identification of genes related to phenylpropanoid biosynthesis in flax

The whole genome shotgun assembly of flax [10] was first surveyed to identify the genes potentially related to phenylpropanoid biosynthesis. BLAST analyses were carried out to search against the 88,420 scaffolds including 43,471 gene models. A total of 69 genes were identified and organized in gene families containing between 3 and 15 members (Table 1) and the presence of the corresponding transcripts for each gene was checked among the different public EST databases. Only *Lus4CL8*, *Lus4CL9* and *LusCCoAOMT5* have no corresponding ESTs (E value <e-50) and the expression of *LusHCT1*, *LusCCR3*, *LusCCR8*, *LusCAD8* and *LusCAD11* was also undetectable when using whole genome microarrays [22]. In addition to these 8 genes, no expression data were obtained for *LusCCR9*, *LusCCR11, LusCCR12* and *LusCAD9* using EST-based microarrays [11, 23]. The intron/exon structure of the genes is graphically represented in Additional file 1: Figure S1.

**Table 1:** Characteristics of the flax phenylpropanoid genes identified in this work

Gene name	Gene model	Genome	Microarray EST	GenBank EST	Ref
*LusPAL1*	Lus10040416	scaffold86	c247	JG065006	5
*LusPAL2*	Lus10023531	scaffold1216	c247	EH791565	4
*LusPAL3*	Lus10026518	scaffold617	c904	JG216766	5
*LusPAL4*	Lus10013805	scaffold618	c904	JG216766	5
*LusC4H1*	Lus10034449	scaffold310	c1220	JG081734	5
*LusC4H2*	Lus10019110	scaffold30	c32549	JG085936	1;5
*LusC4H3*	Lus10021671	scaffold208	c6570	GW864597	2
*LusC4H4*	Lus10035011	scaffold43	c4373	JG075414	1;5
*LusC4H5*	Lus10027598	scaffold2	c11079		
*Lus4CL1*	Lus10026143	scaffold319	c3640	CA483243	
*Lus4CL2*	Lus10008677	scaffold1635	c3640	CA483243	
*Lus4CL3*	Lus10024123	scaffold353	c680	JG220816	5
*Lus4CL4*	Lus10005390	scaffold547	c680	JG218659	5
*Lus4CL5*	Lus10002791	scaffold125	c15833	JG239276	5
*Lus4CL6*	Lus10016135	scaffold344	c3980	EH791222	4
*Lus4CL7*	Lus10021431	scaffold612	c3980	GW866203	2
*Lus4CL8*	Lus10025842	scaffold605			
*Lus4CL9*	Lus10038259	scaffold28			
*LusHCT1*	Lus10002321	scaffold120		JG213400	5
*LusHCT2*	Lus10026097	scaffold319	c3481	JG235206	5
*LusHCT3*	Lus10010786	scaffold18	c6233	JG202486	5
*LusHCT4*	Lus10026123	scaffold319	c8096	CV478884	2
*LusHCT5*	Lus10022163	scaffold342	c6233	JG202486	5
*LusC3’H1*	Lus10033524	scaffold701	c581	JG230193	5
*LusC3’H2*	Lus10020847	scaffold711	c5820	JG226878	5
*LusC3’H3*	Lus10020850	scaffold711	c5820	CA483318	
*LusCCoAOMT1*	Lus10027888	scaffold1143	c395	JG228418	5
*LusCCoAOMT2*	Lus10002837	scaffold810	c395	JG228418	5
*LusCCoAOMT3*	Lus10019841	scaffold1491	c2503	JG094269	5
*LusCCoAOMT4*	Lus10014074	scaffold1247	c2503	JG231505	3;5
*LusCCoAOMT5*	Lus10034584	scaffold9			
*LusCCR1*	Lus10041651	scaffold272	c5445	JG227337	5
*LusCCR2*	Lus10024068	scaffold353	c2262	GW866594	2
*LusCCR3*	Lus10008774	scaffold729		JG203738	5
*LusCCR4*	Lus10022239	scaffold225	c11788	GW865040	2
*LusCCR5*	Lus10024138	scaffold353	c13273	JG149727	5
*LusCCR6*	Lus10042399	scaffold123	c20639	JG103050	5
*LusCCR7*	Lus10026273	scaffold898	c20639	JG103050	5
*LusCCR8*	Lus10006885	scaffold329		JG097906	5
*LusCCR9*	Lus10003780	scaffold806		JG088940	5
*LusCCR10*	Lus10012930	scaffold434	c6761	JG215733	5
*LusCCR11*	Lus10030973	scaffold261		JG240727	5
*LusCCR12*	Lus10035369	scaffold151		JG240727	5
*LusF5H1*	Lus10028361	scaffold413	c772	JG214534	5
*LusF5H2*	Lus10041811	scaffold272	c772	JG214534	5
*LusF5H3*	Lus10014273	scaffold275	c2179	GW865908	2
*LusF5H4*	Lus10025975	scaffold319	c2179	GW867449	2
*LusF5H5*	Lus10034300	scaffold310	c56836		
*LusF5H6*	Lus10012582	scaffold6	c56836		
*LusF5H7*	Lus10022303	scaffold225	c56836		
*LusF5H8*	Lus10041511	scaffold272	c56836		
*LusCOMT1*	Lus10015576	scaffold233	c2253	JG229091	5
*LusCOMT2*	Lus10032929	scaffold51	c629	JG229091	5
*LusCOMT3*	Lus10009442	scaffold981	c4476	GW865137	2
*LusCAD1*	Lus10027864	scaffold1143	c2456	JG020400	5
*LusCAD2*	Lus10002812	scaffold810	c2456	JG020400	5
*LusCAD3*	Lus10019811	scaffold1491	c3049	GW865020	2
*LusCAD4*	Lus10014104	scaffold1247	c3049	JG215109	5;6
*LusCAD5*	Lus10014363	scaffold275	c4852	JG019640	5
*LusCAD6*	Lus10010149	scaffold587	c6705	GW867690	2
*LusCAD7*	Lus10026070	scaffold319	c4852	JG102096	5
*LusCAD8*	Lus10023268	scaffold98		JG062652	5
*LusCAD9*	Lus10035956	scaffold76		EH792205	4
*LusCAD10*	Lus10002089	scaffold575	c4394	JG255907	5
*LusCAD11*	Lus10025706	scaffold605		GW866334	2
*LusCAD12*	Lus10002302	scaffold120	c5057	JG282203	5
*LusCAD13*	Lus10002300	scaffold120	c6631	JG216469	5
*LusCAD14*	Lus10009955	scaffold200	c3693	JG140771	5
*LusCAD15*	Lus10039595	scaffold15	c3758	JG217534	5

1: PR Babu, KV Rao and VD Reddy [86]); 2: A day, M Addi, W Kim, H David, F Bert, P Mesnage, C Rolando, B Chabbert, G Neutelings and S Hawkins [87]); 3: A day, G Neutelings, F Nolin, S Grec, a Habrant, D Cronier, B Maher, C Rolando, H David, B Chabbert, et al. [30]); 4: MJ roach and MK Deyholos [23]); 5: P Venglat, D Xiang, S Qiu, SL stone, C Tibiche, D cram, M Alting-Mees, J Nowak, S Cloutier, M Deyholos, et al. [88]); 6: M Wrobel-Kwiatkowska, M Starzycki, J Zebrowski, J Oszmianski and J Szopa [60])

### In silico and expression approaches to identify monolignol biosynthetic genes

**Fig. 2:**
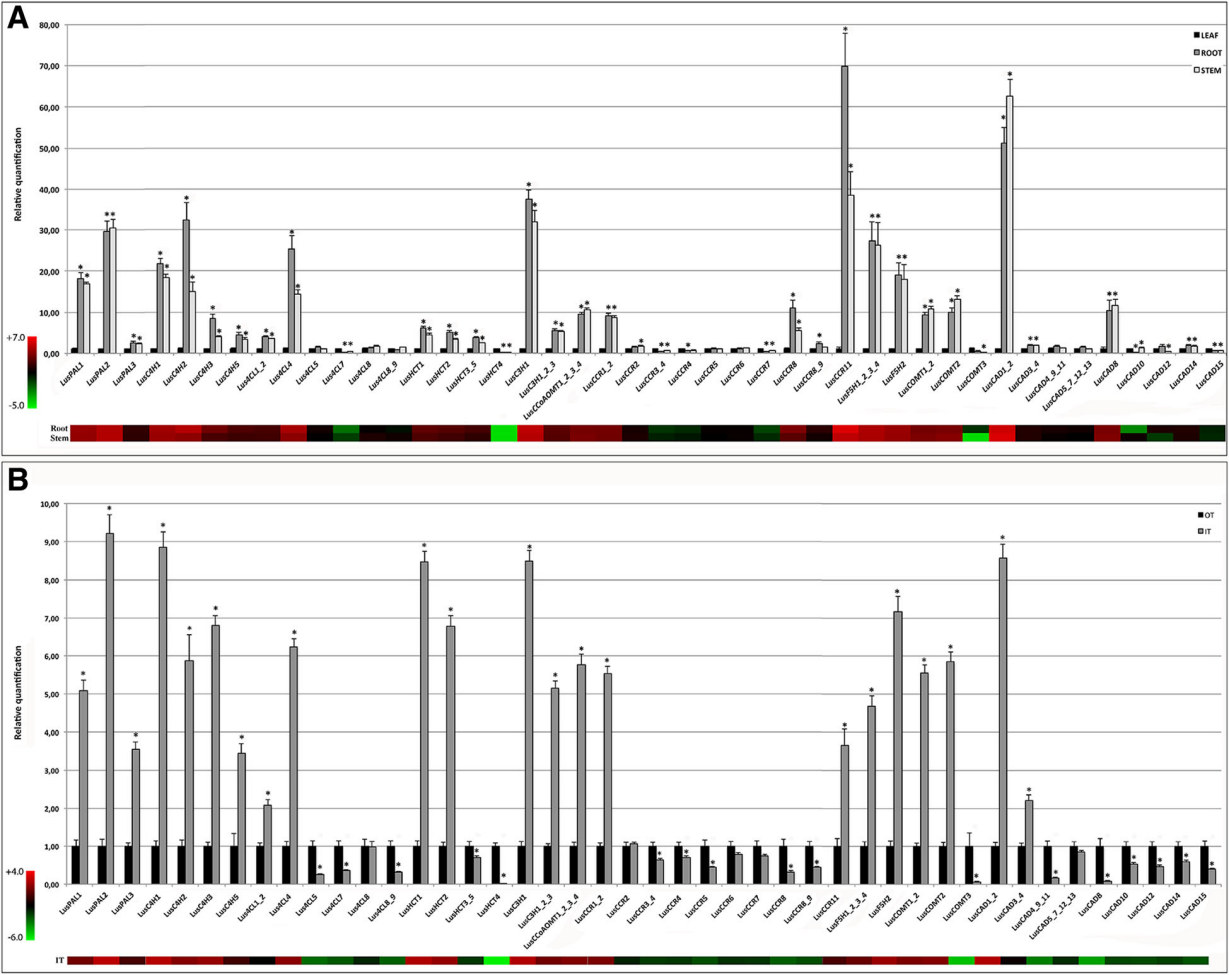
Expression of the flax phenylpropanoid genes determined by HT-RT-qPCR. **a** gene expression in leaves, roots and whole stems. **b** gene expression in the inner xylem-rich tissues (IT) and outer fiber-rich stem tissues (OT) of the stem. Values are means ± SD (*n* = 9). Asterisks indicates values that were determined significantly different from their control (**a**: Leaf and **b**: OT) using a Mann & Whitney’s test (* *P* < 0.01). The heat maps under the histograms represent the expression values in the roots and stems compared to the leaves (**a**) or in the IT compared to the OT (**b**). The colour codes and relative expression ranges in log2 values are represented on the left. * = significant difference in expression

To identify the genes implicated in lignin precursor biosynthesis, a phylogenetic analysis was first performed by combining the protein sequences derived from the flax phenylpropanoid genes with those from *Arabidopsis thaliana*, *Vitis vinifera* and *Populus trichocarpa* (Additional file 2: Figure S2). Different *bona fide* proteins characterized at the biochemical level or by forward/reverse genetic approaches previously used to identify lignin genes in *Eucalyptus grandis* [24] were also added to the data. In addition to this in silico sequence comparison, we also performed HT-RT-qPCR on whole stems, roots and leaves as well as on inner stem xylem-rich tissues and outer stem bast fiber-rich tissues (Fig. 2). Gene expression was also determined in leaves and whole stems under different stress conditions (Additional file 3: Figure S3). Due to the high conservation of several gene sequences and intron/exon positions in some clades, but also the lack of annotation of the more specific 5′/3′-UTRs portions in the genome, we sometimes had to design primers targeting several close-related genes. Taken together, we performed amplifications on 35 single, 12 double, 2 triple and 4 quadruple gene groups (Additional file 4: Table S1). In our experimental conditions, we were unable to detect the expression of *LusPAL4*, *LusC4H4*, *Lus4CL3, Lus4CL6, LusCCR10*, *LusCCR12*, *LusF5H5*, *LusF5H6*, *LusF5H7*, *LusF5H8* and *LusCAD6* genes whatever the organs or environmental conditions. We then used both the phylogenetic analyses (Additional file 2: Figure S2) and the HT-RT-qPCR (Fig. 2) data to identify the potential lignin-specific genes in flax. The major conclusions are summarized in the following subchapters.

#### PAL

The deamination step catalysed by the PAL enzymes leads to the formation of cinnamic acid, which may further undergo a series of esterification, hydroxylation and methylation steps. There are no clear reports on a single “lignin-specific” *PAL* gene in the usual plant models such as *Arabidopsis* or *Populus* because of a probable functional redundancy between the members of this gene family. For this reason, it was not easy to highlight *PAL* genes that are essential for lignin biosynthesis in flax when using only a phylogenetic approach. The 4 flax *PAL* genes identified in this work were all located among the *bona fide* genes. Although the bootstrap values were sometimes low among the subdivisions in this part of the tree, *LusPAL1* and *LusPAL2* were closely related to the 3 xylem-specific poplar genes *PtrPAL2*, *PtrPAL4* and *PtrPAL5* [25] while *LusPAL3* and *LusPAL4* were closer to the *Arabidopsis* orthologs *AthPAL1* and *AthPAL2* involved in lignin metabolism [26]. *LusPAL1* and *LusPAL2* are highly expressed in stems and roots compared to leaves. Their respective expression levels in the inner stem (xylem tissues) are close to five- and nine-fold higher than that observed in the hypolignified external stem tissues. Consequently, *LusPAL1* and *LusPAL2* are the most likely candidates for lignin biosynthesis in flax.

#### C4H

C4H (CYP73) is the first of the three cytochrome P450 monooxygenases involved in the hydroxylation of phenylpropanoid precursors. In flax, five distinct genes located on different scaffolds were annotated by KEGG as trans-cinnamate 4-monooxygenases (K00487). In the neighbour joining tree, 2 distinct clades were present, one contained all of the *bona fide* functionally characterized orthologs, together with an additional *Vitis* sequence and the flax proteins LusC4H1–4. In the other clade, LusC4H5 was located with PtrC4H3 and the eucalyptus EgrC4H1, suggested to be the main C4H involved in lignin biosynthesis [24] even though *EgrC4H2* was also preferentially expressed in the xylem. The HT-RT-qPCR profiles of the flax genes showed that *LusC4H1*, *LusC4H2, LusC4H3* and *LusC4H5* were expressed in lignified tissues with highest levels for *LusC4H1–2* making them the best candidates for a major role in lignification.

#### 4CL

The 4CL enzymes produce specific CoA thioesters of 4-hydroxycinnamic acids at an important crossroad in the phenylpropanoid pathway. Nine *4CL* gene models were identified in the genome of *Linum usitatissimum*. When the major known sequences were assembled in the phylogenetic tree, a delimited *bona fide* clade containing two separated classes was identified as shown in previous studies [27, 28]. The class I, predicted to be involved in monolignol biosynthesis, contained four flax proteins Lus4CL1–4. The *Lus4CL3* gene remained undetectable in our conditions whereas the other three genes had higher expression in roots and in stems. The relative expression of *Lus4CL4* was 6 fold higher in the xylem as compared to the hypolignified external stem tissues and 15- and 25- fold higher in the stems and roots as compared to the leaves and so is likely involved in lignin biosynthesis.

#### HCT

In the lignin biosynthetic pathway, HCTs first catalyse the formation of *p*-coumaroyl shikimate from *p*-coumaroyl CoA and shikimic acid. To produce caffeoyl CoA, the product is first hydroxylated by C3’H and then converted again by HCT acting in the reverse direction. The closely related acyltransferase hydroxyl-cinnamoyl CoA:quinate hydroxycinnamoyl transferase (HQT) can lead to the formation of chlorogenic acid by the transfer of quinic acid on the same *p*-coumaroyl CoA substrate, forming p-coumaroylquinic acid. The distinction between HCT and HQT genes without further biochemical characterization is not always very easy because of their very similar sequences. We identified five different genes in the flax genome closely related to the previous identified HCT/HQT genes. The expression profiles show that *LusHCT1* and *LusHCT2* are active in the roots and highly expressed in stem-internal tissues. Their position among the *bona fide* HCT sequences in the phylogenetic tree also suggests their implication in lignin biosynthesis. The *LusHCT4* gene is also among the true HCTs but the expression of this gene is approximately 50 fold times higher in the hypolignified external tissues of the stem.

#### C3’H

The C3’H genes generally belong to small hydroxylase subfamilies involved in monolignol biosynthesis. The enzyme (CYP98A3) catalyses the 3′-hydroxylation of 4-coumaroyl shikimate and 4-coumaroyl quinate into the corresponding caffeoyl-conjugated form. In flax, we identified only 3 gene models corresponding to C3’H. A gene duplication event possibly occurred because *LusC3’H2* and *LusC3’H3* are both located on the same scaffold in a 14 kb region. When considering the structure (Additional file 1: Figure S1) and homology between the 3 genes, it is possible to speculate that a first duplication event of *LusC3’H1* formed the *LusC3’H2* gene, which in turn was duplicated forming *LusC3’H3*. This gene was shorter at the 3′-end but still had between 92 and 98% amino acid sequence identity with the 2 former corresponding proteins. In *Arabidopsis* only one *C3’H* gene was identified whereas an expansion of this family by lineage-specific tandem duplications in *Populus* [29] and *Eucalyptus* [24] was described. The *LusC3’H1* gene is more strongly expressed in the stem and root tissues and therefore probably involved in lignin biosynthesis.

#### CCoAOMT

Among both methylation enzyme families, CCoAOMTs can transfer a –CH3 group from a donor to a hydroxycinnamoyl CoA ester, namely caffeoyl CoA. Among the 5 genes identified in this work, *LusCCoAOMT4* was previously characterized in knockdown flax plants [30]. The four genes *LusCCoAOMT1–4* have high sequence identity, similar gene structures and could not be individualized by the HT-RT-qPCR approach. The amplicon was detected at high levels in lignin-containing organs such as stems and roots compared to leaves and preferentially in the internal stem tissues. In the phylogenetic tree, they collocate with the sequences identified as *bona fide* enzymes in *Arabidopsis*, *Eucalyptus*, *Nicotiana* and *Populus*.

#### CCR

This enzyme is important because it catalyses the first specific step of the monolignol production by reducing hydroxycinnamoyl CoA esters. Taking into account the large size of this gene family as determined by genome sequencing, they are likely to be involved in numerous other metabolic pathways. In flax, we identified 12 genes with very different intron/exon patterns. The *LusCCR3*, *LusCCR5* and *LusCCR6* genes do not fit the 3 patterns described for *Arabidopsis* [31]. Although most of the genes encode proteins with similar sizes, *LusCCR3* appears smaller and thus may be truncated. In *Arabidopsis*, 12 *CCR* genes were present in duplicated chromosome regions including 4 genes distributed in tandem [31]. No similar situation exists in flax although *LusCCR2* and *LusCCR5* were located on the same scaffold but at a distance of 360 kb. The differences in the structure of these 2 genes are however not in favour of a recent duplication event. The *bona fide* lignin biosynthesis genes were grouped in a common clade also containing *LusCCR1, LusCCR2*, *LusCCR11* and *LusCCR12*. The expression profiles of these four genes in the lignified tissues showed that *LusCCR1* and *LusCCR11* are the best lignin-associated candidates.

#### COMT

As indicated by the enzyme name, the caffeate/5 hydroxyferulate *O*-methyl-transferase was first thought to act as a bifunctional enzyme able to transfer a methyl group from S-adenosyl methionine to the C_3_ and C_5_ positions of the aromatic rings of caffeic and 5-hydroxyferulic acids [32]. In fact, the COMT enzyme promotes the biochemical pathway leading to the formation of sinapyl alcohol by catalysing the methylation of 5-hydroxyconiferyl alcohol [33]. Only 3 gene models were identified within the flax *COMT* gene family, which is low compared to many other species [34]. *LusCOMT1* and *LusCOMT2* have close exon/intron patterns but are present on different scaffolds. They both are expressed at higher rates in lignified tissues. The position of the corresponding proteins within the *bona fide* group in the phylogenetic tree seems to support the hypothesis of their implication in the lignin biosynthetic pathway.

#### F5H

The F5H cytochrome P450-dependent monooxygenase is required for the hydroxylation of coniferaldehyde and coniferyl alcohol leading to the formation of S lignin through the synthesis of sinapyl alcohol [35]. In flax, 8 gene models were identified and compared to orthologous models expressed in lignin-rich tissues. In the phylogenetic tree, these proteins are located on a branch separated from the *bona fide* enzymes by a node predicted by a low bootstrap value. The flax proteins were further subdivided into 2 groups of sequences with similar exon/intron patterns. The first group contained *F5H1*, *F5H2*, *F5H3* and *F5H4,* which could not clearly be separated by HT-RT-qPCR during the design of the primers except for *F5H2*. The common *F5H1_2_3_4* and specific *F5H2* amplicons were detected at high levels in lignin-containing organs such as roots and whole stems and also in xylem-rich outer tissues. The second group with *F5H5*, *F5H6*, *F5H7* and *F5H8* genes had undetectable expression.

#### CAD

The NADPH-dependent CAD enzymes play an important role in monolignol biosynthesis by catalyzing the reduction of hydroxycinnamyl aldehydes into their corresponding monolignols which is the last step in the biosynthetic pathway [36]. They are encoded by a multigenic family and their homologs have been detected widely in bacteria and eukaryota but not in animals [37]. Classification by several authors highlighted 3 to 7 groups/classes based on phylogenetic approaches [38–41]. Recently an accurate classification proposed a separation in 3 functional clades for both dicots and monocots [42] with a lignin specific class I and a possible lignin-associated class II. In the present study we identified 15 flax gene models predicted in the published genome. Both *LusCAD12* and *LusCAD13* were located on scaffold120 in a 5.5 kb fragment and have the same exon/intron pattern associated with a high (84%) amino acid sequence identity, reflecting a possible duplication event. *LusCAD1*, *LusCAD2*, *LusCAD3* and *LusCAD4* segregate with the known *bona fide* genes within the phylogenetic tree. Because of their very high sequence identity (98,6% at the protein level), it was not possible to separate *LusCAD1* and *LusCAD2* when designing the primers for qRT-PCR. Their expression profiles show that the amplicons were mostly present in the lignified tissues such as roots and whole stems and also in xylem-rich outer tissues. To a lesser extent, *LusCAD8* showed the same expression profile in these organs but revealed an opposite behaviour when comparing outer and inner stem tissues. This gene is located outside the *bona fide* clade.

### Cell−/tissue-specific expression of lignin and selected lignin biosynthetic genes

**Fig. 3:**
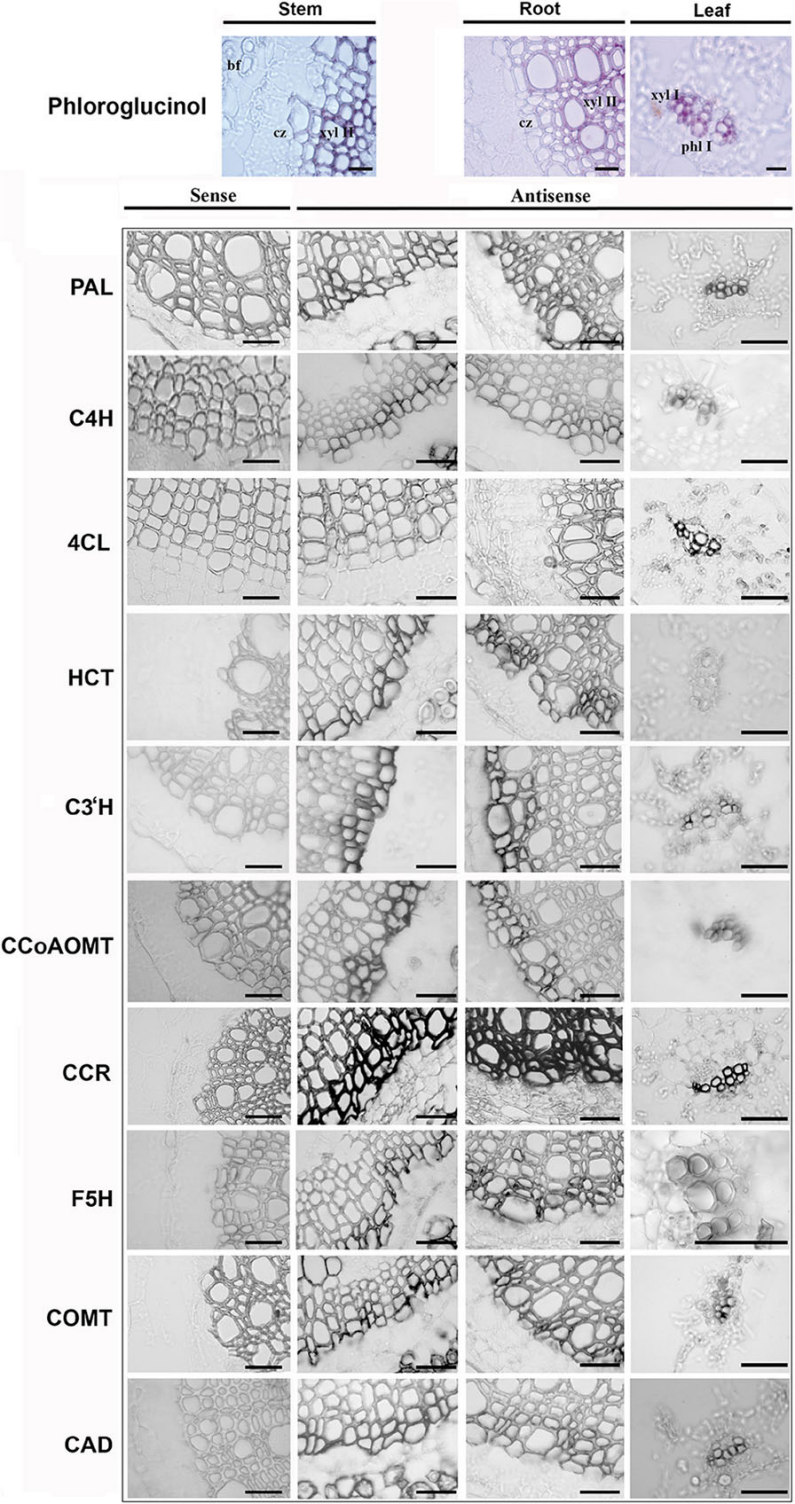
Lignin and lignin gene transcript localization by in situ hybridization in flax stems, roots and leaves. A set of paraffin stem sections (10 μm-thick) were first hybridized with the corresponding sense probes (left column) in order to confirm the specificity of the signal. Bar = 50 μm. The top panels show lignin localization (in red) by phloroglucinol-HCl staining. Phl I: primary phloem; xyl I: primary xylem; xyl II: secondary xylem; cz: cambial zone; bf: bast fiber. Bar = 50 μm

The likely implication of identified candidate genes in the flax lignification process was further confirmed using in situ hybridization that established a close spatial correlation between lignifying cells determined by the Wiesner reaction and gene expression in stems, roots and leaves (Fig. 3 and summarized in Additional file 5: Table S2). These genes were *LusPAL1*, *LusC4H2*, *Lus4CL4*, *LusHCT1*, *LusC3’H1_2_3*, *LusCCoAOMT1_2_3_4*, *LusCCR1_2, LusF5H1_2_3_4*, *LusCOMT1_2* and *LusCAD1_2*. For most genes, the strongest expression was observed in the stems and the roots in the first 2–5 secondary xylem cell layers from the cambial zone towards the inner part of the organs. In the leaves, the stain was in each case restricted to the primary xylem. For all genes, signal intensity was generally higher in stems and roots compared to leaves except for *CCR* for which equal intensity was observed in all 3 organs. In addition, the *4CL4* probe failed to reveal the presence of the corresponding transcripts in the stems and roots despite the clear organ specificity revealed by the HT-RT-qPCR results. The observed lack of signal might be due to poor probe efficiency since several fragments of the *4CL4* gene were tested but did not produce a positive signal in roots or stems. Overall, these results confirm that the expression of the selected genes is intimately associated with the actively differentiating secondary xylem zone thereby confirming their probable implication in the lignification process.

### Identification of laccase genes and their regulation by microRNAs

**Fig. 4:**
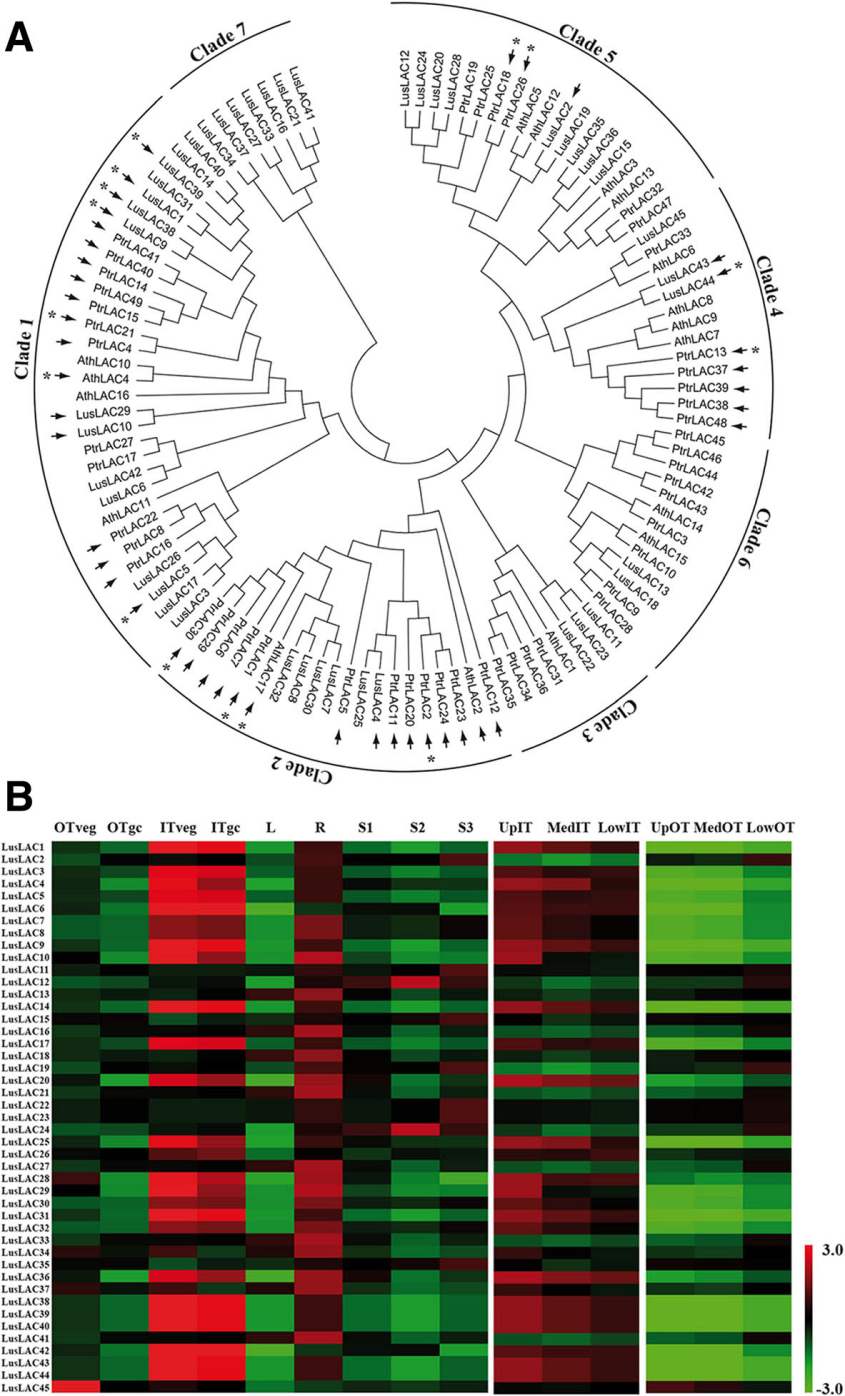
Laccase gene family in flax. **a** A phylogenetic tree was produced in MEGA 6 using the maximum likelihood method based on the JTT matrix-based model. The analysis was performed with 45 flax, 17 *Arabidopsis*, and 49 poplar laccase sequences. The arrows indicate the targets predicted by psRNAtarget and the asterisks, the experimentally validated targets. **b** Heat map showing laccase gene expression in different flax organs and tissues

Laccases play an important role in controlling lignin polymerization by catalysing the oxidation of the precursors [43]. Both in silico prediction and high throughput sequencing in different species [20, 21] have shown that laccase transcript levels are regulated by microRNAs. We identified 45 corresponding gene models in flax and used them to construct a phylogenetic tree (Fig. 4a). In comparison to previous published data reporting the classification of laccases in 6 clades [21], our tree contained an additional clade with 7 flax sequences. The expression profiles (Fig. 4b) of these laccase genes were extracted from public high throughput transcriptomic data [11, 15]. Of the 45 genes, 21 showed higher expression in ‘high lignin’ inner tissues compared to ‘low lignin’ outer tissues and are preferentially expressed on the top part of the stem where the development of the SCW takes place.

**Fig. 5:**
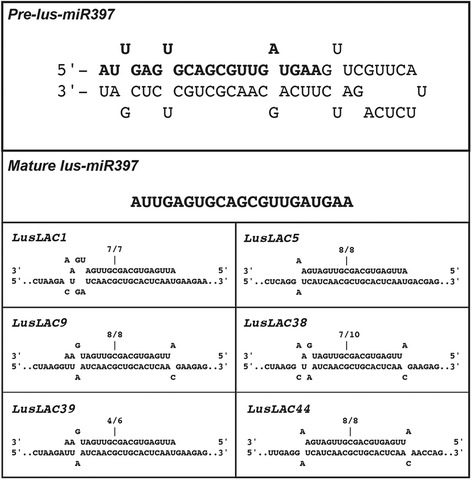
*Lus-miR397* structure and validation of predicted targets. The sequences of the *pre-lus-miR397* and the mature *lus-miR397* are indicated. The association with laccase targets are shown only for the six transcripts validated by RLM 5′-RACE. The cleavage sites are indicated above the corresponding position and the number of cloned RACE products sequenced is shown above each sequence (number of sequenced fragments at this position/total number of sequenced clones)

**Fig. 6:**
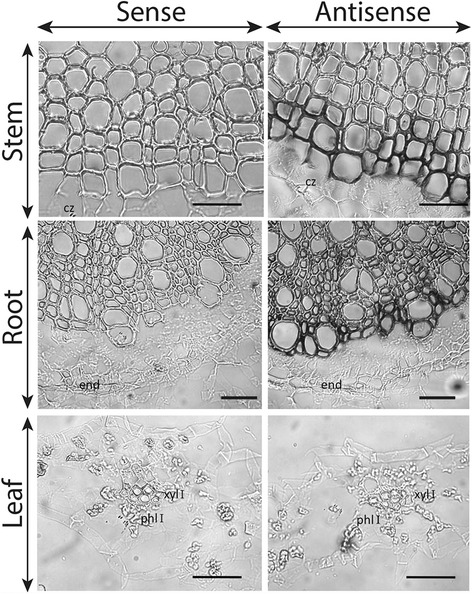
Spatial localization of *lus-mir397* transcripts in flax organs. Paraffin sections (10 μm) of the stems, roots and leaves were hybridized with the sense controls (left) and the antisense specific probes (right). cz: cambial zone; end: endodermis; phl I: primary phloem; xyl I: primary xylem. Bar = 20 μm

Based on previous studies reporting predictive and experimental data on the cleavage of *LAC4* and *LAC17* transcripts in *Arabidopsis* [20] and potentially 29 laccase transcripts in *Populus* [21] by *miR397*, we first searched for a flax orthologous miRNA precursor sequence in the EST and genome databases as previously described [44]. The c3244 EST from the genolin database [11] and scaffold1999 extracted from the phytozome genomic database [45] both contained the pre-miRNA sequence (Fig. 5). The deduced mature *lus-miR397* sequence was first used to search for targets among the flax laccases and among these, 11 genes were predicted with penalty mismatches included between 0 and 2.5 within the duplex. The amplification and sequencing of the 5′-end of the degraded transcripts by RLM-RACE allowed us to experimentally validate the cleavage of *LusLAC1*, *LusLAC5*, *LusLAC9*, *LusLAC38*, *LusLAC39* and *LusLAC44* among the predicted targets (Fig. 5). These 6 genes were present among the 21 genes highly expressed in the xylem-rich tissues. The five first laccase transcripts are close to the *Arabidopsis* lignin-related *AthLAC4* gene [43] and all belong to the clade 1 (Fig. 4A). *LusLAC44* belongs to clade 4 including *PtrLAC13* which was shown to be cleaved by *Ptr-miR397* [46]. These results demonstrate that flax laccase genes are regulated by microRNAs suggesting that this mechanism is implicated in controlling lignification in this species. Further evidence supporting this idea was provided by in situ hybridisation data (Fig. 6) indicating a close correlation between tissue−/cell-specific expression of the *lus-miR397* precursor, monolignol specific genes and lignified tissues in stems and roots.

### Stress-related flax phenylpropanoid gene expression

Since lignin is an important component in stress responses, we analysed the expression of the phenylpropanoid genes with a special focus on the previous identified lignin-associated genes to search for those regulated by environmental modifications. Flax plants and isolated organs were submitted to a range of different stress conditions and gene expression determined by HT-RT-qPCR. The expression profiles were shown in Additional file 3: Fig. S3 and summarized in Table 2. Salicylic acid (SA) plays an important role in plant defense signaling and accumulates in response to pathogen infection [47]. In flax stems, SA significantly down-regulated the lignin-related genes *LusC4H1* and *LusCCoAOMT1_2_3_4* but had an opposite effect on *LusPAL2* and the *LusF5H1_2_3_4* group. Higher expression levels were also observed for *Lus4CL8* and *LusCAD10*. In parallel, SA had a moderate effect on the expression of lignin genes in treated leaves. Only the up-regulation of the *LusCAD4_9_11* group and *LusCAD8* were evident. Methyljasmonate is a precursor of the active jasmonate form in plants [48] and mimics biotic as well as abiotic stresses [49]. MeJA application led to a very strong down-regulation of most of the phenylpropanoid genes including all of the identified *bona fide* lignin genes within the leaves except for *LusCCR11*. On the contrary, this signal regulator has a low overall effect on gene expression in flax stems. It is also interesting to note that some genes show no detectable expression levels in either the presence or absence of SA and MeJA, possibly due to the in vitro conditions used for organ incubation.

**Table 2:** Summary of the stress response of flax phenylpropanoid genes

Gene	Stress								
	SA		MeJA		Drought		Wound		Light
	Stem	Leaf	Stem	Leaf	Stem	Leaf	Stem	Leaf	Stem
LusPAL1*				−	−		+		−
LusPAL2*	+			−	−		+	−	
*LusPAL3*	−			−	−			+	
*LusPAL4*									
LusC4H1*	−		−	−		+	+	+	
LusC4H2*				−	+		+		
*LusC4H3*	−	+					+		
*LusC4H4*									
*LusC4H5*									−
*Lus4CL1_2*				−		+	+	+	
*Lus4CL3*									
Lus4CL4*				−	−		+		−
*Lus4CL5*	+	−	−	−	+		+	+	+
*Lus4CL6*									
*Lus4CL7*				−	−		−		
*Lus4CL8*	+			−					
*Lus4CL8_9*	+	−		−				+	
LusHCT1*				−	−		+		
LusHCT2*				−	−		+	−	
*LusHCT3_5*	+	−		−	+	+	+	+	+
*LusHCT4*			−	−			−	−	
*LusHCT5*									
LusC3’H1*				−	−		+		+
*LusC3’H1_2_3*				−	−		+		
LusCCoAOMT1_2_3_4*	−			−	−				
*LusCCoAOMT5*									
LusCCR1_2*				−	−				
*LusCCR2*		−		−					+
*LusCCR3_4*						+			
*LusCCR4*						+			
*LusCCR5*	+		−	−	+	+	+		+
*LusCCR6*			−	−					
*LusCCR7*			−	−					
*LusCCR8*				−					
*LusCCR8_9*				−				+	+
*LusCCR10*									
LusCCR11*					+	+	+		
*LusCCR12*									
LusF5H1_2_3_4*	+		+	−	+		+	+	
*LusF5H2*	+		−	−	+		+	+	
*LusF5H5*									
*LusF5H6*									
*LusF5H7*									
*LusF5H8*									
LusCOMT1_2*				−	+		+	+	
LusCOMT2*	+			−				+	
*LusCOMT3*	−			−	+				
LusCAD1_2*				−	−		+	+	
*LusCAD3_4*		+	−	−	+		+		
*LusCAD4_9_11*	−	+	−	+	+		+		
*LusCAD5_7_12_13*				−					+
*LusCAD6*									
*LusCAD8*	−	+	−		+	+		+	
*LusCAD10*	+			−	+	+	+		
*LusCAD12*		−		−	+		+	+	
*LusCAD14*					+		+	+	+
*LusCAD15*	+			−	+		+		+

Asterisks indicate potential lignin-specific flax genes

The effect of abiotic stresses was further tested by inducing water deficit on whole flax plants grown in soil. Under these conditions, *LusC4H1* and *LusCCR11* were the only lignin-associated genes showing higher expression in leaves while most of the other *bona fide* genes were slightly upregulated in the stems. The effect of wounding was also tested on flax stems and leaves. In these organs, the effect on the lignin-associated genes was much stronger in the stems compared to the leaves. The modulation of phenylpropanoid gene expression was also evaluated after 48 h of continuous light illumination. When compared to 16 h/8 h light/dark conditions, only the *LusPAL1* and *Lus4CL4* genes were downregulated and 7 genes or gene-groups were significantly upregulated. When taken together, our results showed that the strongest impact on the previously genes identified as involved in lignin biosynthesis, occurred in the stem during dehydration, wounding and in the presence of SA.

### Gene clustering

**Fig. 7:**
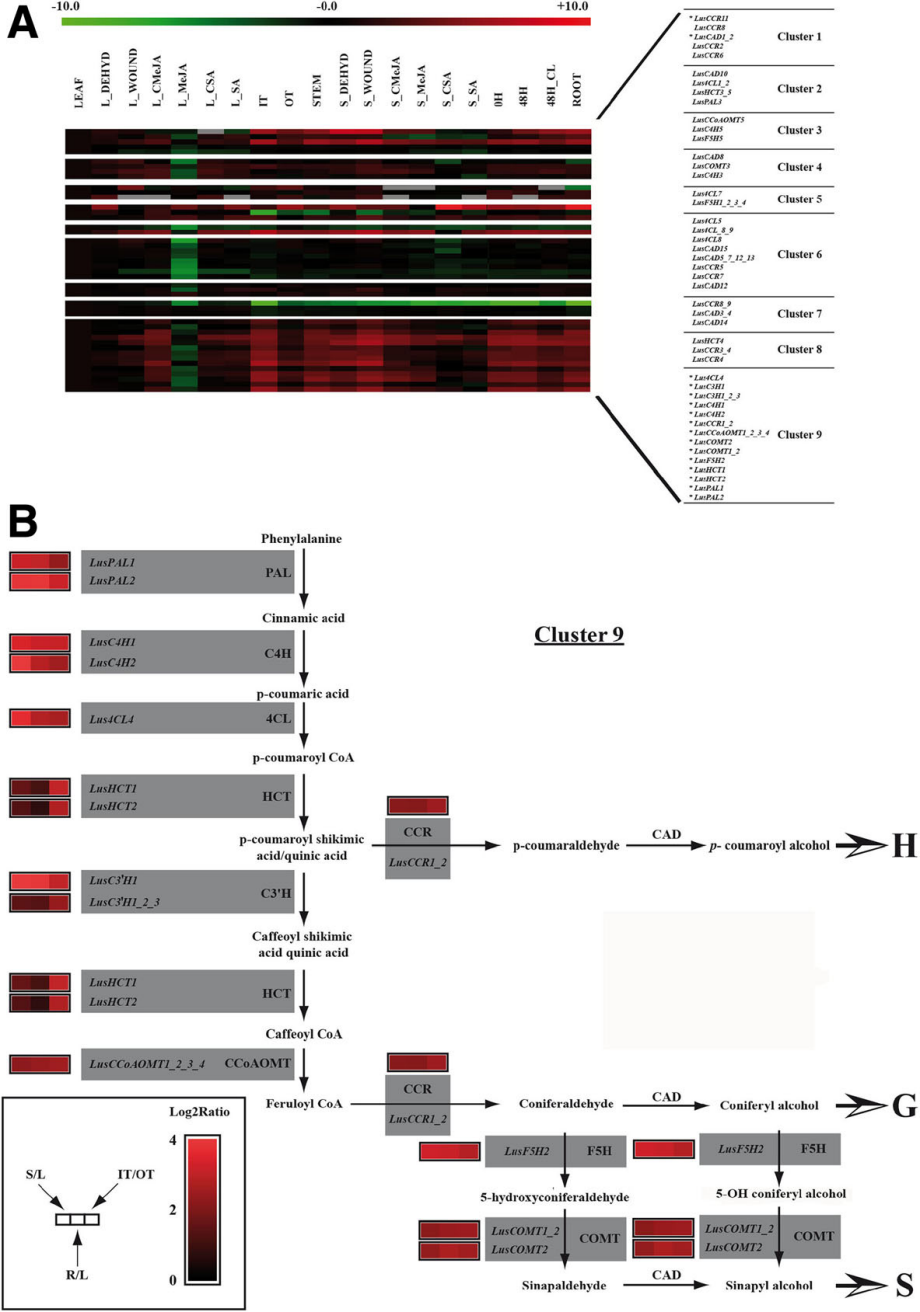
Clustering of flax phenylpropanoid genes according to their expression profile in different organs and stress conditions. **a** the k-means medians clustering function from the TIGR MultiExperiment Viewer (TM4 Mev v4.8.1) was used to cluster the genes according to their expression profiles. The distance metric was Pearson correlation and the default parameters were used for the k-means calculation. The expression profiles are represented as heat maps. The colour code and expression range in log2 values are represented on top of the figure. **b** the biosynthetic pathway leading to monolignol production and further to H, G and S units is summarized. Three expression ratios are indicted as heat maps beside the genes. C: control; CL: continuous light; DEHYD: drought stressed stems; IT: internal stem tissues; L: leaf; MeJA: methyljasmonate; OT: outer stem tissues; R: root; S; stem; SA: salicylic acid

Genes involved in the same biological process are often co-regulated. To further confirm the implication of the former identified genes in the lignification pathway, we searched for possible common expression profiles among the gene set. A k-means clustering approach was used to identify correlated groups exhibiting similar expression profiles in the different tissues and/or stress conditions. The number of flax genes in each cluster ranged between 2 and 14 (Fig. 7a). Among the flax phenylpropanoid associated genes, all the potential *bona fide* genes identified previously, except for *LusCAD1_2* and *LusCCR11*, were included in cluster 9 (Fig. 7b) showing that the expression of these genes were closely co-regulated in flax. Altogether, the clustering results provide further support for the involvement of the identified genes in lignification.

Gene co-regulation requires the activity of common transcription factors and since MYB proteins are known to function as master switches of secondary cell wall and lignin gene transcription [50, 51] we examined the promoters of our identified genes for MYB consensus motifs named MBS (MYB binding site) (C/T)AAC(A/T)A(A/C)C and MBSIIG (or SMRE: secondary wall MYB-responsive element) (C/T)ACC(A/T)A(A/C)C. Our results (Additional file 6: Table S3) showed that the MBSIIG site was present in the 500 bp proximal promoter fragments of all the *bona fide* genes present in the cluster 9 whereas both MBS sites are absent from the *LusCCR11* promoter in cluster 1. Interestingly, both MYB sites were absent from the proximal promoters of the *LusF5H1_2_3_4* genes which is in accordance with previous results showing that *F5H* is directly regulated by a NAC SND1 transcription factor [52].

## Discussion

### Identification of genes involved in the lignin biosynthetic pathway in flax

In the past 25 years, a tremendous amount of information about lignin biosynthesis has been obtained using model plants such as *Arabidopsis* and *Populus* species. Currently, the possibility to obtain comprehensive genomic sequences in other species is allowing the scientific community to gather additional data on other species. Secondary cell walls (SCWs), whether they are specialized in water conduction (xylem) or mechanical support (sclerenchyma), usually contain around 25% lignin. However, in some specialized tissues such as bast fibers, the cell wall contains very low amounts of lignin together with high cellulose levels [53, 54]. Although these cells only contain low amounts of lignins, the presence of these phenolic molecules can have an important impact on the quality of the final products such as linen tissues and biocomposites.

In this context, we carried out a comprehensive genome-wide analysis of the gene set involved in phenylpropanoids, and more specifically in lignin biosynthesis in flax. The size of individual phenylpropanoid gene families is difficult to compare between species due to differences in the stringency of the sequence annotations. This is especially true for widespread enzymatic activities such as hydroxylases or methyltransferases. On the other hand, gene expression profiling often gives clear information about the function of the more specific *bona fide* lignin genes [24]. Usually, individual qRT-PCR experiments are performed to validate lignin transcript level pattern analysis [55] but HT-RT-qPCR approaches are increasingly used as they are more appropriately sized for characterizing the members of multigene families in a single step [56]. In flax, we first identified the genes potentially involved in lignification by examining the expression profiles in roots and stems compared to the leaves and also by comparison between the internal xylem stem tissues (high lignin) and the outer fiber-bearing tissues (low lignin).

Further confirmation of the likely implication of selected phenylpropanoid genes in lignification was provided by in situ hybridization, allowing the precise localization of the lignin biosynthetic gene expression. Both in situ hybridization and also promoter-reporter gene studies conducted on a low number of genes previously showed that *bona fide* lignin genes were expressed in the first few cell layers of differentiating secondary xylem [17–19, 57, 58]. To our knowledge, our work reports for the first time an in situ hybridization approach on a large number of lignin genes in a higher plant species. As was shown by the results of the high throughput expression analyses, the very high expression levels in the xylem-rich tissues compared to the bast fiber cells is in accordance with our previous results suggesting that lignification of flax fibers is at least in part regulated at the transcriptional level [15].

Finally, when the expression patterns of all the phenylpropanoid genes were considered, most of these lignin-associated genes were found to be co-expressed within a same cluster confirming that these genes were indeed involved in a common metabolic pathway. The presence of common MYB *cis*-element motifs in the promoters of these genes associated to their co-expression was already described for the orthologous genes in *Arabidopsis* [59]. In this species, MYB58 can directly activate most lignin biosynthesis genes but not *F5H* showing that the syringyl lignin biosynthesis is activated by a different regulatory pathway probably controlled by NAC transcription factors [52].

### Selection of candidates for lignin engineering in flax

The success of targeted engineering of cell wall lignin genes to improve the quality of different flax-derived products depends upon the correct identification of individual family gene members involved in the trait to be modified. Some genes potentially involved in lignin biosynthesis have already been partially characterized in flax. The first flax lignin cDNA sequence was deposited in the GenBank database in 2005 and the corresponding *CCoAOMT* gene functionally characterized [30]. It was chosen because it is responsible for the synthesis of feruloyl CoA used for the formation of both G- and S-lignin units and, as confirmed by down-regulation, because it plays a central role in maintaining SCW integrity by regulating the quantity and the S/G proportion in the non-condensed lignin fraction. This gene corresponds to the *LusCCoAOMT4* identified in our study and is included in the *bona fide* group of genes expressed in lignified tissues. In another study, a *CAD* gene fragment sharing 100% homology with *LusCAD4* was targeted by a downregulation strategy [60]. This gene associated with *LusCAD3* was not retained in our work as a major lignin gene because of its much lower differential expression between lignified and poorly-lignified organs or tissues when compared to *LusCAD1_2*. Although the RNAi transformed plants contained lower amounts of lignins compared to the control, it is interesting to note that M Wrobel-Kwiatkowska, M Starzycki, J Zebrowski, J Oszmianski and J Szopa [60]) did not observe the typical brown-midrib phenotype of *CAD* mutants observed in other species. In contrast, TILLed flax chemical mutants of *LusCAD1* possess the characteristic orange-coloured xylem phenotype [16] confirming the likely involvement of this gene in the lignification process. Flax lignin has also received interest because of the very low amounts of S-units [53] so we were therefore interested to examine the *F5H* gene family. This enzyme is specifically required for the synthesis of S lignin since it catalyses the 5-hydroxylation of coniferaldehyde and/or coniferyl alcohol [35]. Its down-regulation in *Arabidopsis* leads to a reduced S/G value [61]. The expression ratio of *LusF5H2* in the lignified organs and tissues is comparable to those of other identified genes so the biosynthesis of low amounts of S units in flax does not seem to be controlled by *F5H* gene expression. However, further computational modelling and biochemical characterization of the members of the F5H family have to be done in order to provide more clues on the specificity of these enzymes towards various substrates.

### *lus-miR397* controls several laccase genes in flax

Laccases are mono- or multimeric copper-oxidases in eukaryotes and procaryotes with a broad range of aromatic or non-aromatic substrates [62] and have been implicated in the oxidative polymerization of monolignols. Among the 17 gene models described in *Arabidopsis*, *AthLAC4*, *AthLAC11* and *AthLAC17* were clearly shown to contribute to the constitutive lignification of the floral stems [43, 63]. In *Populus trichocarpa*, 49 gene models have been identified but it was not yet possible to clearly identify those involved in lignification [21]. In flax, we identified 45 laccase gene models of which 23 were highly expressed in lignified tissues. MicroRNA targeting (*miR397*) of some laccase transcripts, including *AthLAC4* and *AthLAC17*, contribute to the overall gene regulation [20, 21]. This microRNA has been identified in many gymnosperms, monocots and dicots with 1 to 3 copies per species [64]. In this study, we experimentally demonstrated that transcripts corresponding to six flax laccase genes were targeted by *lus-miR397*. On the basis of our phylogenetic analysis, five of these genes were found in the clade 1, which also contained *AthLAC4* whereas six non-targeted genes were present in clade 2 along with *AthLAC17* showing that either no functional ortholog of this gene is present in flax or that these genes have lost their ability to be cleaved by *lus-miR397*. Strong evidence for a likely role of microRNA regulation of laccase-mediated lignification in flax was provided by the cell−/tissue-specific localization of the corresponding transcript precursors. Our results are in agreement with those obtained with a *GUS*-*GFP* fusion driven by the *miR397b* promoter, showing that this gene is expressed in the stem vascular tissues of *Arabidopsis* [65]. Further analyses should allow a better understanding of whether *miR397* plays a role in maintaining constant transcript levels or is implicated in a finer control of different laccase paralogs.

### Stress related phenylpropanoid genes

Flax plants selected for fiber production are mostly cultivated around the Mediterranean and temperate climate zones. In the context of climate change, cell wall metabolism, structure and fiber quality are likely to be affected in the future and it is therefore informative to know which genes are affected by biotic or abiotic stresses. We took advantage of the high throughput gene expression approach to try and distinguish between developmental lignin genes and stress-related phenylpropanoid gene expression.

Defence-related hormones such as SA and MeJA can control lignin gene activation. SA is an important signalling molecule for systemic acquired resistance [66] and triggers the expression of lignin genes in many different species [67]. In flax, several lignin genes showed opposite expression profiles in the presence of SA suggesting that the composition of lignin may change during the defence mechanism or that more specific metabolic pathways including these genes are activated. The effect of MeJA on this metabolic pathway seems more subtle. This component has been shown to participate in the signal transduction pathway in response to different forms of stresses [68]. A dose effect was described in *Fragaria* fruits [69] as well as an opposite *CAD* gene expression pattern in tea plants [70]. In our experimental conditions, most of the flax genes were underexpressed indicating that the synthesis of lignin is probably not part of the response to this elicitor. Abiotic stresses can also have an influence on lignification. Mechanical stresses can be responsible for the deposition of various polymers including lignin that may function as a physical barrier around the injury zone [71]. In this context, lignin genes are activated in different species including sweet potato [72], maize [73] or *Arabidopsis* [74]. Most of the flax lignin genes were activated in the stem but less in the leaves showing that in this species, lignin is also synthesized in reaction to wounding. Outsides these *bona fide* genes, several common phenylpropanoid genes including *CADs* were also activated in both organs showing that they may potentially be involved in the production of other abiotic stress responsive metabolites as was already proposed in poplar [38]. The effect of drought stress on plants is more difficult to understand because it can have positive or negative roles on lignification depending on the species, the organs/tissues and the intensity and duration of the stress period [75]. In our experimental conditions (12 days after watering stop) most of the lignin genes were upregulated in the stems and the leaves. The increase in lignification has been described as one of the reactions included in a general adaptation strategy of plants faced with water loss and may result in an increase of mechanical strength and/or water impermeability [76].

## Conclusion

We have identified, in this study, the individual members of the multigenic families most likely involved in lignin biosynthesis (and polymerisation) in flax. These data provide the knowledge base necessary to undertake targeted engineering and/or marker-based selection of lignin biosynthesis in flax, as well as important information on the behaviour of these genes in a number of different stress conditions. The latter information will be particularly useful for farmers and breeders in the current context of increasing variability in weather conditions associated with climate change.

## Methods

### Plant material

For HT-RT-qPCR expression analysis, flax plants (*Linum usitatissimum* L. cv Diane) were grown in 12 × 12 cm soil-containing pots in a greenhouse under 16 h/20 °C day and 8 h/18 °C night conditions for 35 days. For developmental expression studies, (i) leaves, ii) 2-cm root fragments taken under the stem base, (iii) top 4 cm of stem, (iv) fiber-rich outer-tissues and (v) xylem-rich inner-tissues from stem base were harvested. For stress expression studies, the following samples were taken from these 35-day-old plants: (i) leaves and stems harvested 24 h after wounding with a scalpel, (ii) leaves and stems incubated for 24 h in Petri dishes containing 200 μM salicylic acid (SA) or methyljasmonate (MeJA) dissolved in ½ MS medium [77], (iii) leaves and stems from plants submitted to a drought stress imposed by withholding water for 12 days, (iv) stems from plants first placed in phytotrons during 7 days for acclimation and then submitted to continuous light for 48 h at 20 °C. All the samples were triplicates and always frozen immediately in liquid nitrogen before storage at −80 °C prior to RNA extraction. For in situ hybridization, the median stem, leaves and 2-cm root fragments were collected and fixed as described below.

### In silico identification and analysis of phenylpropanoid genes in *L. usitatissimum*

The flax phenylpropanoid protein and gene sequences were identified in the annotated v1.0 of the *Linum usitatissimum* genome hosted on the Phytozome website [45]. The entire database derived from the published genome sequences [10] was queried with the names of the gene families used as keywords and also by BLASTp interrogation with previously lignin-related identified sequences from *Arabidopsis thaliana* [78] and *Populus trichocarpa* [25]. When it was necessary, unknown portions of some sequences were replaced by the corresponding sequences found in public EST databases and truncated sequences were discarded. For each gene model, the presence of a corresponding EST was searched in the dbEST [79] and genolin [11] databases. The intron/exon structures were also retrieved and the identities of the protein sequences compared. The k-means medians clustering function from the TIGR MultiExperiment Viewer (TM4 Mev v4.8.1) was used to cluster the genes according to their expression profiles. The distance metric was Pearson correlation and the default parameters were used for the k-means calculation.

### Phylogenetic analyses

For each gene family, the protein sequences were aligned with ClustalW and the phylogenetic analyses obtained with the MEGA 6.06 [80] software for performed by maximum likelihood method based on the JTT matrix-based model [81]. Consensus trees were generated with 1000 bootstrap replicates.

### HT-RT-qPCR using a 96.96 dynamic array

Total RNA was extracted from the different organs and tissues using the TriReagent method (Molecular Research Center). RNA integrity and concentration were evaluated with RNA Standard Sens Chips in the Experion automated capillary electrophoresis system (Bio-Rad). cDNA was then synthesized using the High Capacity RNA-to-cDNA Kit (Applied Biosystems) according to the manufacturer’s instructions. The large scale quantitative RT-PCR was performed with a BioMark HD System using a Fluidigm 96.96 dynamic array (IntegraGen, Evry France) according to the Fluidigm Advanced Development Protocol with EvaGreen using primer pairs listed in Additional file 4: Table S1. A first preamplification reaction (1 cycle: 95 °C 10 min; 14 cycles: 95 °C 15 s, 60 °C 4 min) was performed for each sample in 10 μl by pooling primer pairs (final concentration, 50 nM), 3.3 μl cDNA, and 5 μl 2X PreAmp Master Mix (Applied Biosystems). For each assay, 5 μl 10X Assay Mix containing 9 μM forward primer, 9 μM reverse primer, and 1X Assay Loading Reagent was loaded into the chip. The following thermal cycles were executed: 1 cycle: 95 °C 10 min; 40 cycles: 95 °C 15 s, 60 °C 1 min. The amplifications on the three biological samples were always conducted in triplicate and were also performed on a mix of cDNAs to determine the amplification efficiencies. The Ct values were analysed with the qBase + software (Biogazelle, Belgium). The data normalization was performed using the previous experimentally validated reference genes *LusETIF5A1*, *LusUBI1* and *LusEF1A* [82].

### Histochemical analysis

Flax stems, roots and leaves were fixed with 4% paraformaldehyde in phosphate buffer (0.2 M, pH 7) for 16 h. They were then washed in phosphate buffer containing 0.1 M glycine and dehydrated through a series of ethanol solutions and progressively embedded in paraffin (ParaplastPlus; Oxford Labware). Tissue sections were obtained with a Leica RM2065 microtome and placed on silanised-coated slides. Before staining, tissue sections were deparaffinised with Histoclear (Labonord) and rehydrated with decreasing concentrations of ethanol. The presence of lignin was determined by staining with the Wiesner reagent (phloroglucinol–HCl) [83].

### Spatial gene expression determination by in situ hybridization

To obtain DNA templates for RNA probe synthesis, PCR amplifications were performed with gene-specific primers tailed with a T7 RNA polymerase binding site Additional file 4: Table S1. The amplicons (1 μg) were used as templates to synthesize sense and antisense probes for each gene, with the incorporation of UTP–digoxigenin as the label using the DIG labelling mix (Roche). Deparaffinised sections were treated as previously described [84], hydridized and probe detected with NBT (nitro-blue tetrazolium chloride)/BCIP (5-bromo-4-3′-indolylphosphate *p*-toluidine salts) solutions according to the manufacturer’s instructions. Controls without probe or with sense probe were performed to check for endogenous alkaline phosphate activity.

### miRNA target prediction and experimental validation

The sequence of the *lus-miR397* precursor was identified among the known flax ESTs using stringent rules described elsewhere [44]. The laccase transcript models were analysed with psRNATarget [85] to predict the targets that can potentially match with the microRNA without any gaps. When referring to the miRNA sequence, there were not more than one mismatch between the nucleotides number 1 and 9, none between 10 and 11, and no more than 2 consecutive mismatches after position 11. The experimental validation of the laccase targets was carried out by using a modified protocol of the RNA ligation mediated (RLM) RACE included in the GeneRacer kit (Invitrogen). RNA was extracted from a mix of flax organs and the cleaved transcripts (without 5′-cap) were selectively ligated to a 5′-RNA adaptor. After reverse transcription, PCR amplification was performed with a 5′ adaptor-specific primer and a reverse gene specific primer Additional file 4: Table S1 located downstream of the predicted cleavage site. The fragments were then gel purified and sequenced.

## Additional files

Additional file 1: Figure S1. Flax phenylpropanoid gene structures. The name of the gene is indicated followed by the length in bp between the start and stop codons in parentheses. The exons are shown as boxes and the introns as lines. The scale in bp is shown on the top of the figure. (DOC 40 kb)

Additional file 2: Figure S2. Molecular phylogenetic analysis of the phenylpropanoid genes. (PDF 1379 kb)

Additional file 3: Figure S3. Expression of the flax phenylpropanoid genes under stress conditions as determined by HT-RT-qPCR. 0H: start of the light period on the first day; 48H: end of the night on the second day; 48H_CL: 48 h after continuous light; CONT: control; Dehyd: dehydration; L: leaf; MeJA: methyljasmonate; S: stem; SA: salicylic acid. (PDF 1906 kb)

Additional file 4: Table S1. Primers used for HT-RT-qPCR, RLM RACE and in situ hybridization. (DOC 76 kb)

Additional file 5: Table S2. Description of the gene expression localization in the tissues of flax roots, stems and leaves. (JPEG 391 kb)

Additional file 6: Table S3. Position of the MBSII and MBSIIG specific *cis* elements on both strands (+/−) of the flax phenylpropanoid gene promoters. (XLSX 15 kb)

## Abbreviations

4CL: 4-coumarate:CoA ligase
C3’H: *p*-coumarate 3-hydroxylase
C4H: cinammate 4-hydroxylase
CAD: cinnamyl alcohol dehydrogenase
CCoAOMT: caffeoyl CoA 3-*O*-methyltransferase
CCR: cinnamoyl CoA reductase
COMT: caffeate/5-hydroxyferulate *O*-methyl-transferase
F5H: ferulate 5-hydroxylase
HCT: hydroxycinnamoyl-CoA:shikimate hydroxycinnamoyl transferase
MeJA: methyljasmonate
PAL: Phenylalanine ammonia-lyase
SA: salicylic acid
