# Supplementary material for: Spatial regulation of monolignol biosynthesis and laccase genes control developmental and stress-related lignin in flax
Source: BMC Plant Biol. 2017 Jul 14;17:124. doi: 10.1186/s12870-017-1072-9 (PMC5513022; doi:10.1186/s12870-017-1072-9)
Supplement: Supplementary file 1 — Flax phenylpropanoid gene structures. The name of the gene is indicated followed by the length in bp between the start and stop codons in parentheses. The exons are shown as boxes and the introns as lines. The scale in bp is shown on the top of the figure. (DOC 40 kb) [file 12870_2017_1072_MOESM5_ESM.doc]

| **Probe** | **Genes** | **Stem** | **Root** | **Leaf** |
| --- | --- | --- | --- | --- |
| *PAL* | *LusPAL1* | 4-5 first cell layers from the cambial zone | 4-5 first cell layers from the cambial zone | Primary xylem |
| *C4H* | *LusC4H2* | 2-3 first cell layers from the cambial zone | 3-4 first cell layers from the cambial zone | Primary xylem |
| *4CL* | *Lus4CL4* | 2-4 first cell layers from the cambial zone | 2-3 first cell layers from the cambial zone | Primary xylem |
| *HCT* | *LusHCT1* | 3 first cell layers from the cambial zone | 3 first cell layers from the cambial zone | Primary xylem |
| *C3H* | *LusC3H1_2_3* | 1-2 first cell layers from the cambial zone | 2-3 first cell layers from the cambial zone | Primary xylem |
| *CCoAOMT* | *LusCCoAOMT1_2_3_4* | 2-3 first cell layers from the cambial zone | 2-4 first cell layers from the cambial zone | Primary xylem |
| *CCR* | *LusCCR1_2* | 3-4 first cell layers from the cambial zone | 3-4 first cell layers from the cambial zone | Primary xylem |
| *F5H* | *LusF5H1_2_3_4* | 2-3 first cell layers from the cambial zone | 2-4 first cell layers from the cambial zone | Primary xylem |
| *COMT* | *LusCOMT1_2* | 2 first cell layers from the cambial zone | 2 first cell layers from the cambial zone | Primary xylem |
| *CAD* | *LusCAD1_2* | 2-3 first cell layers from the cambial zone | 2-3 first cell layers from the cambial zone | Primary xylem |
