# Supplementary figures and images for: Spatial regulation of monolignol biosynthesis and laccase genes control developmental and stress-related lignin in flax
Source: BMC Plant Biol. 2017 Jul 14;17:124. doi: 10.1186/s12870-017-1072-9 (PMC5513022; doi:10.1186/s12870-017-1072-9)

A

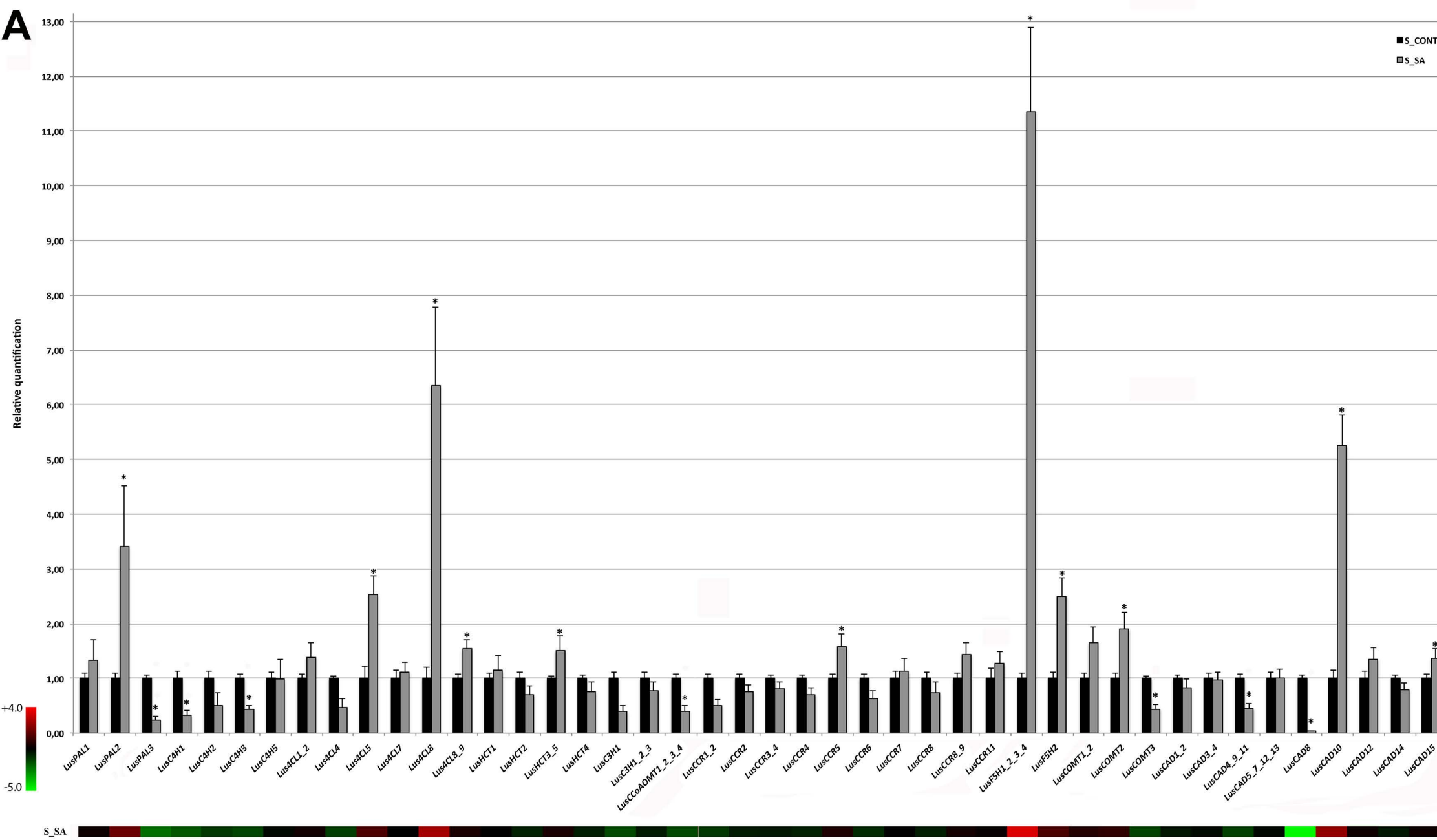

**B**

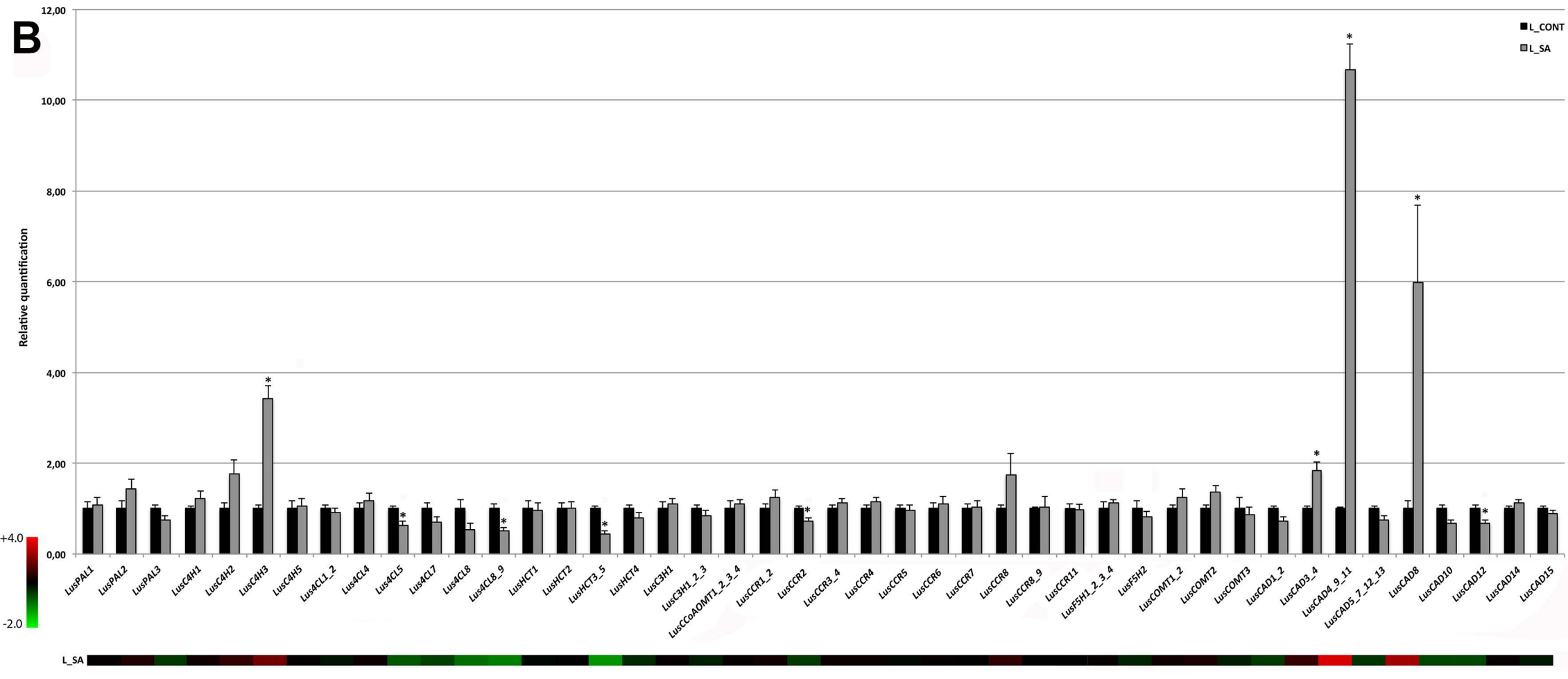

C

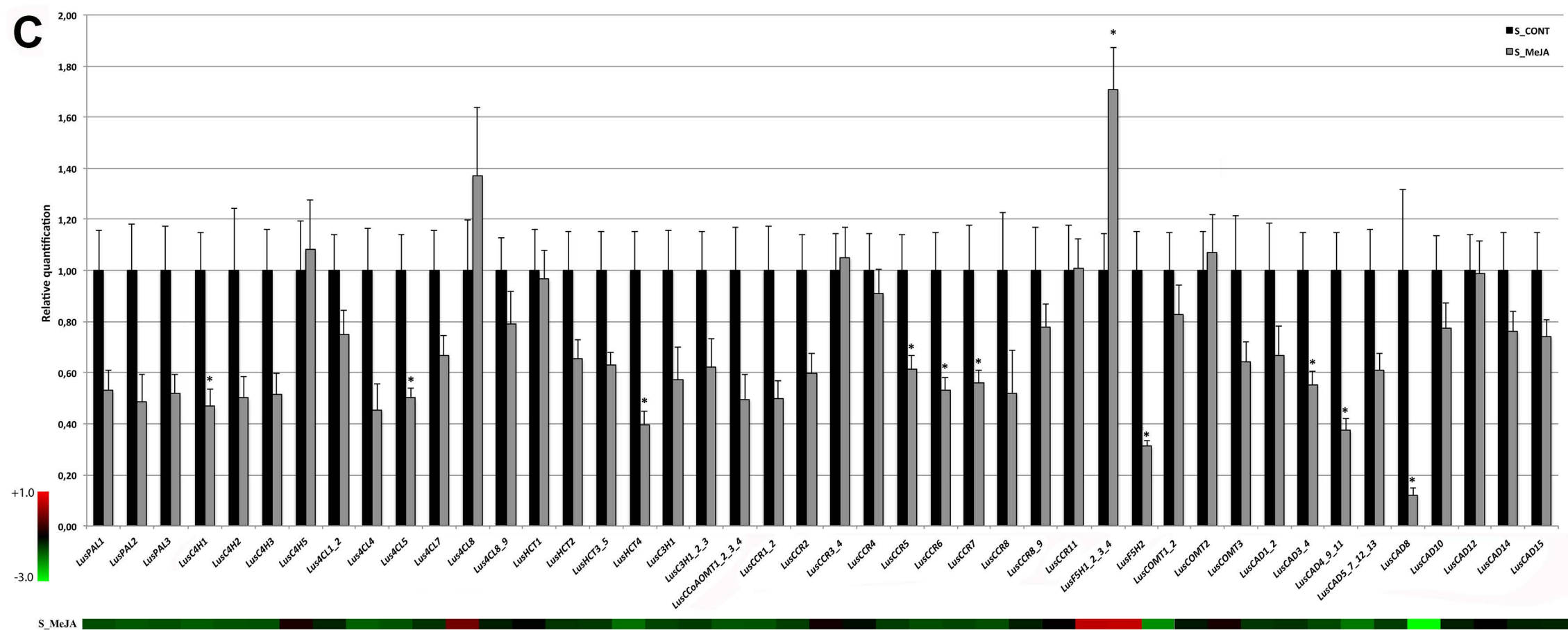

**D**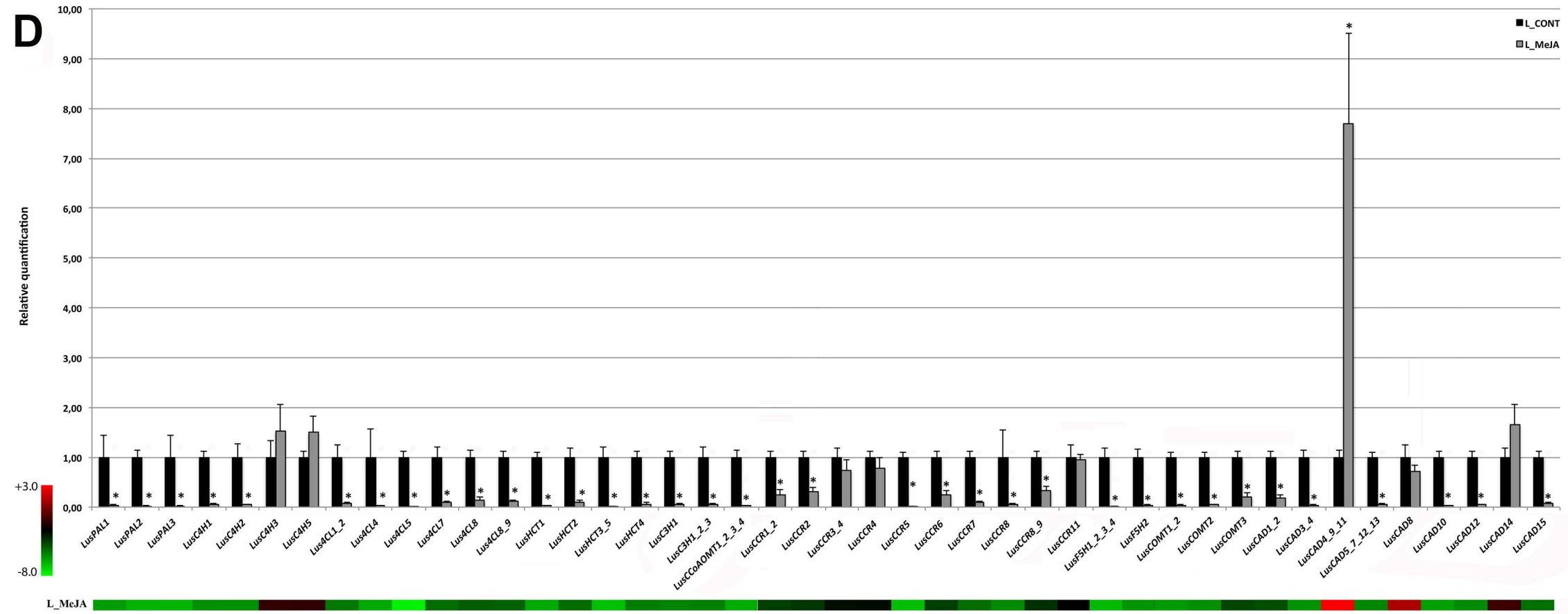

E

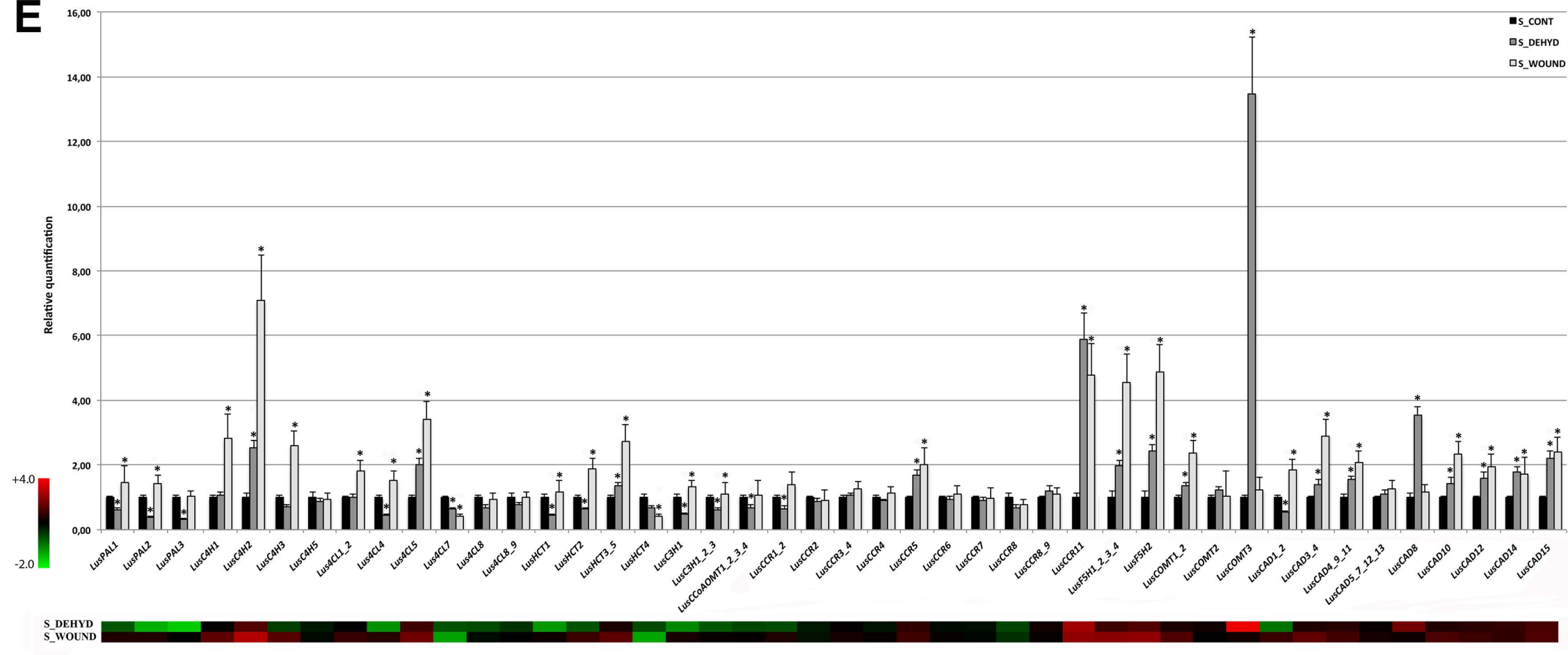

F

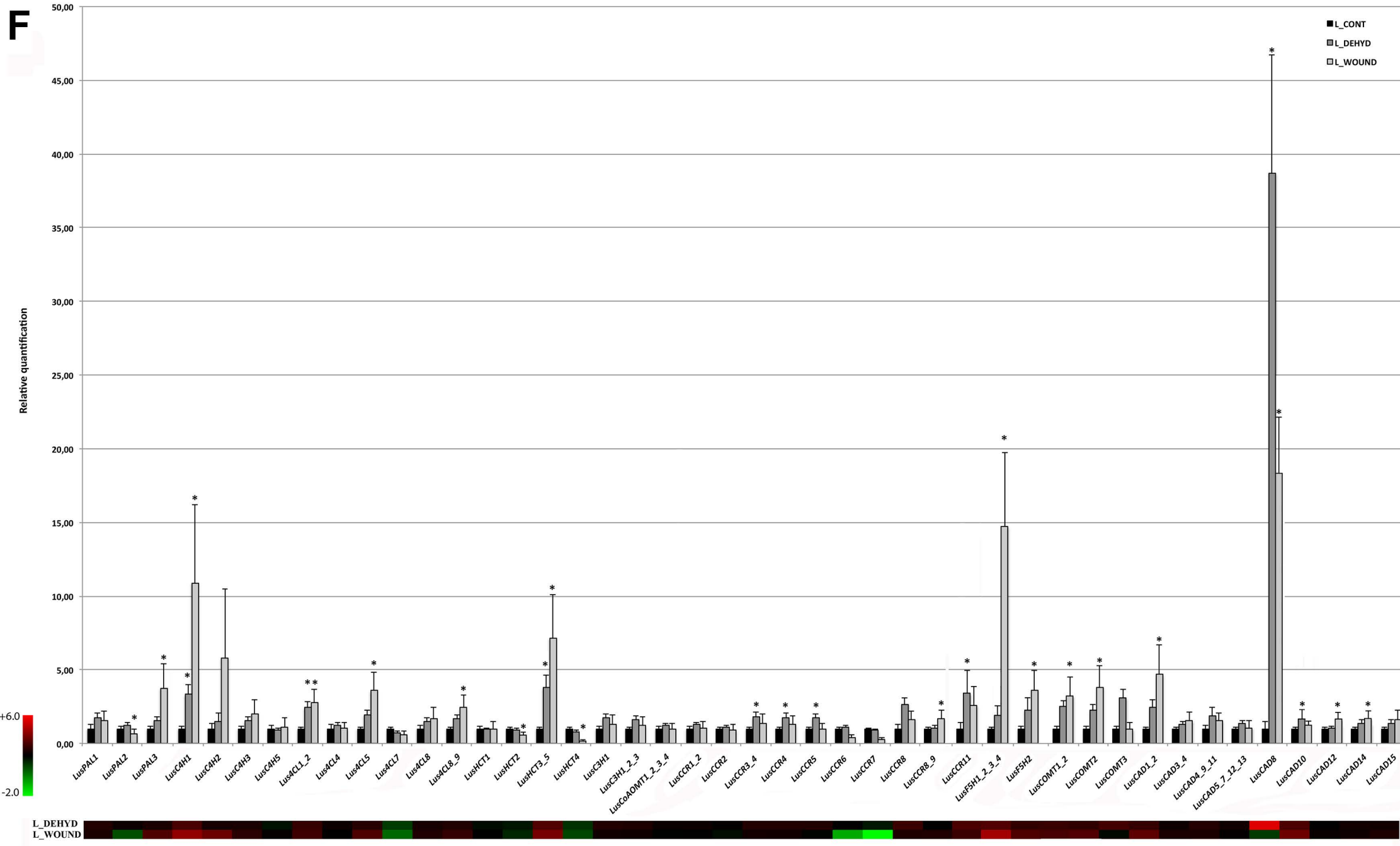

G

Relative quantification

+4.0  
-3.048H  
48H\_CL0H  
48H  
48H\_CL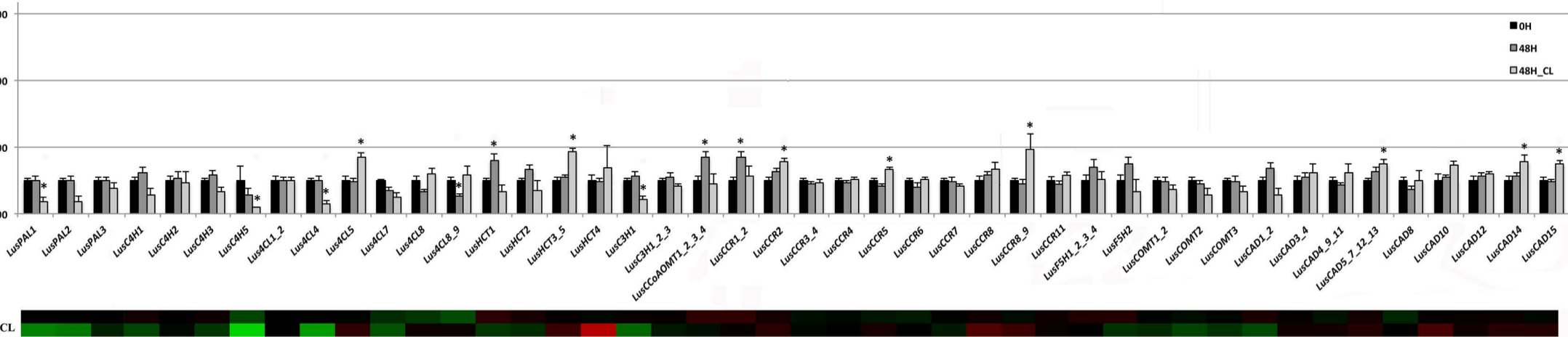

Supplement: Supplementary file 2 — Molecular phylogenetic analysis of the phenylpropanoid genes. (PDF 1379 kb) [file 12870_2017_1072_MOESM3_ESM.pdf]

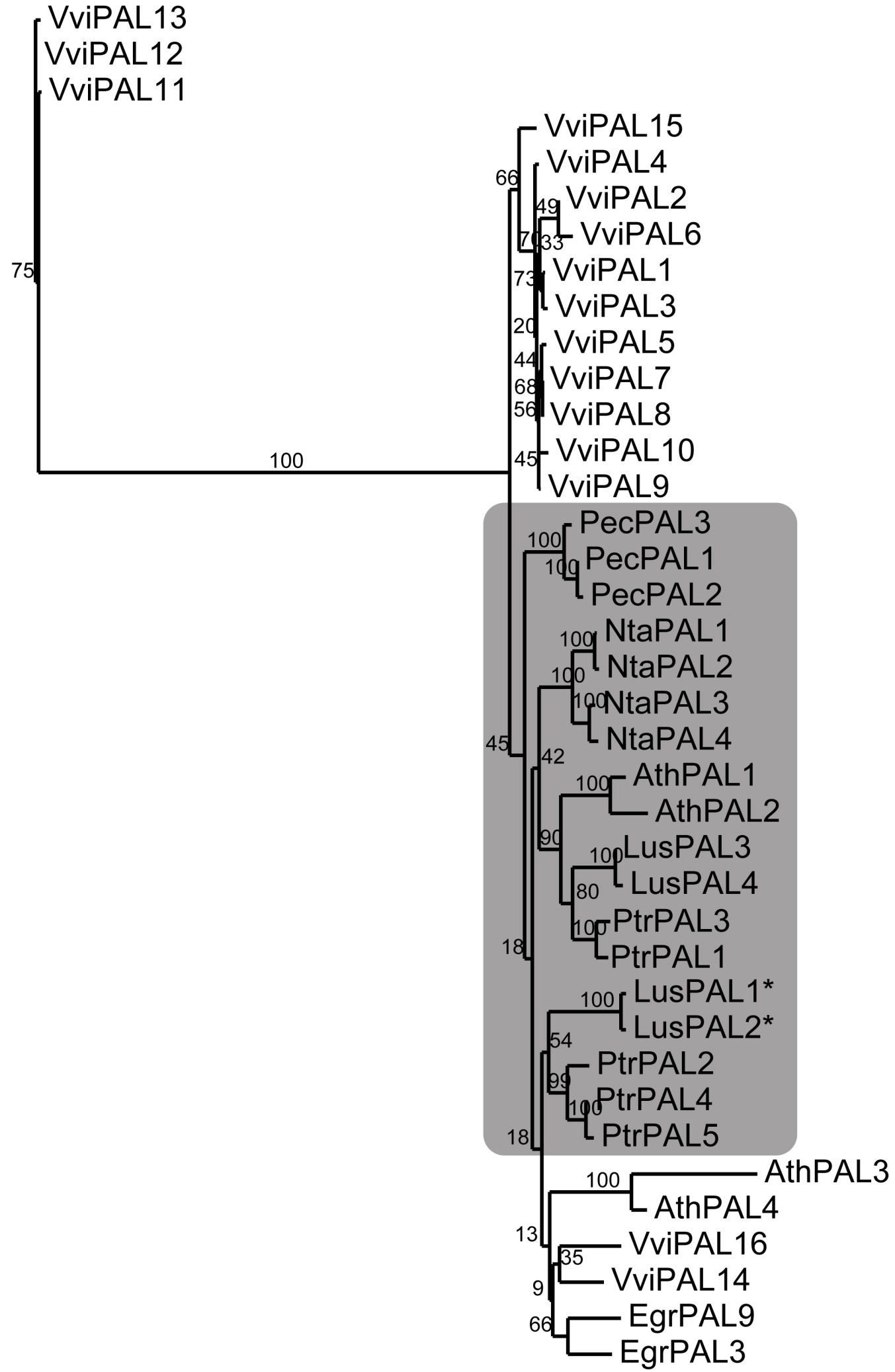

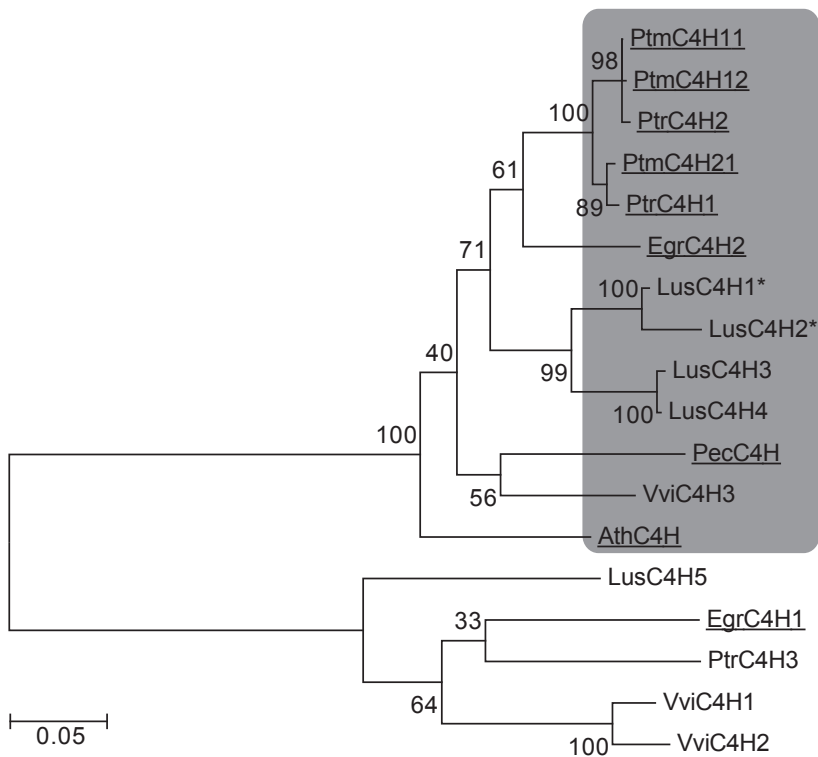

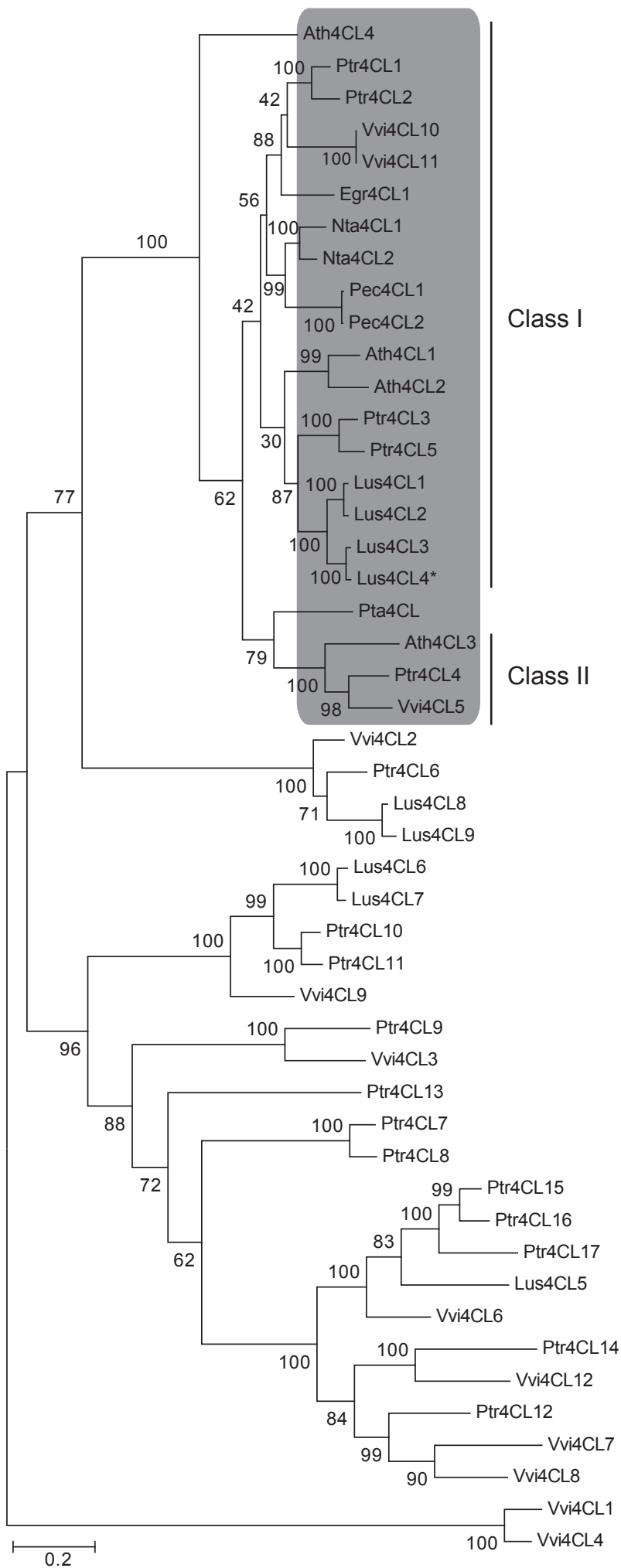

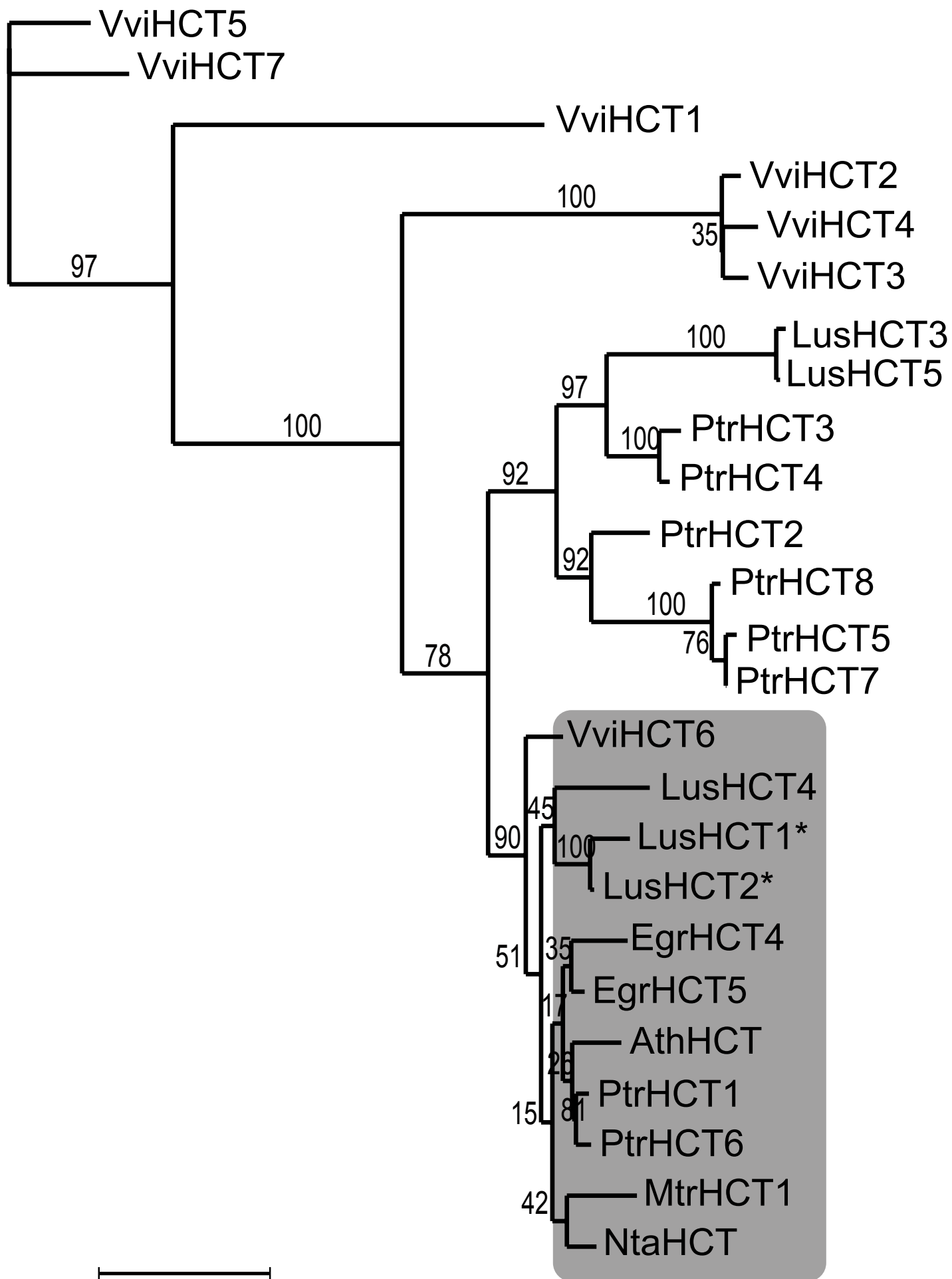

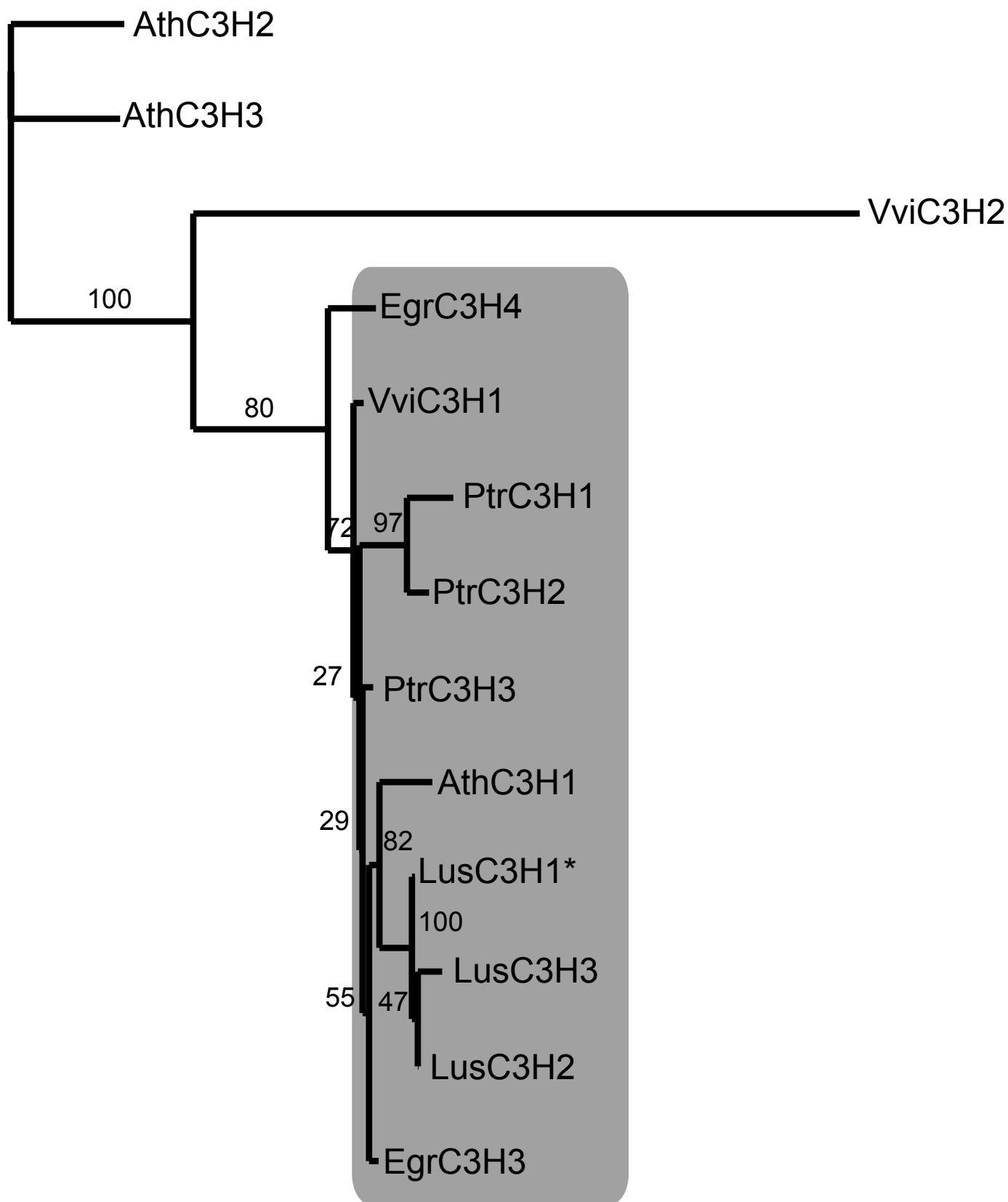

0.6

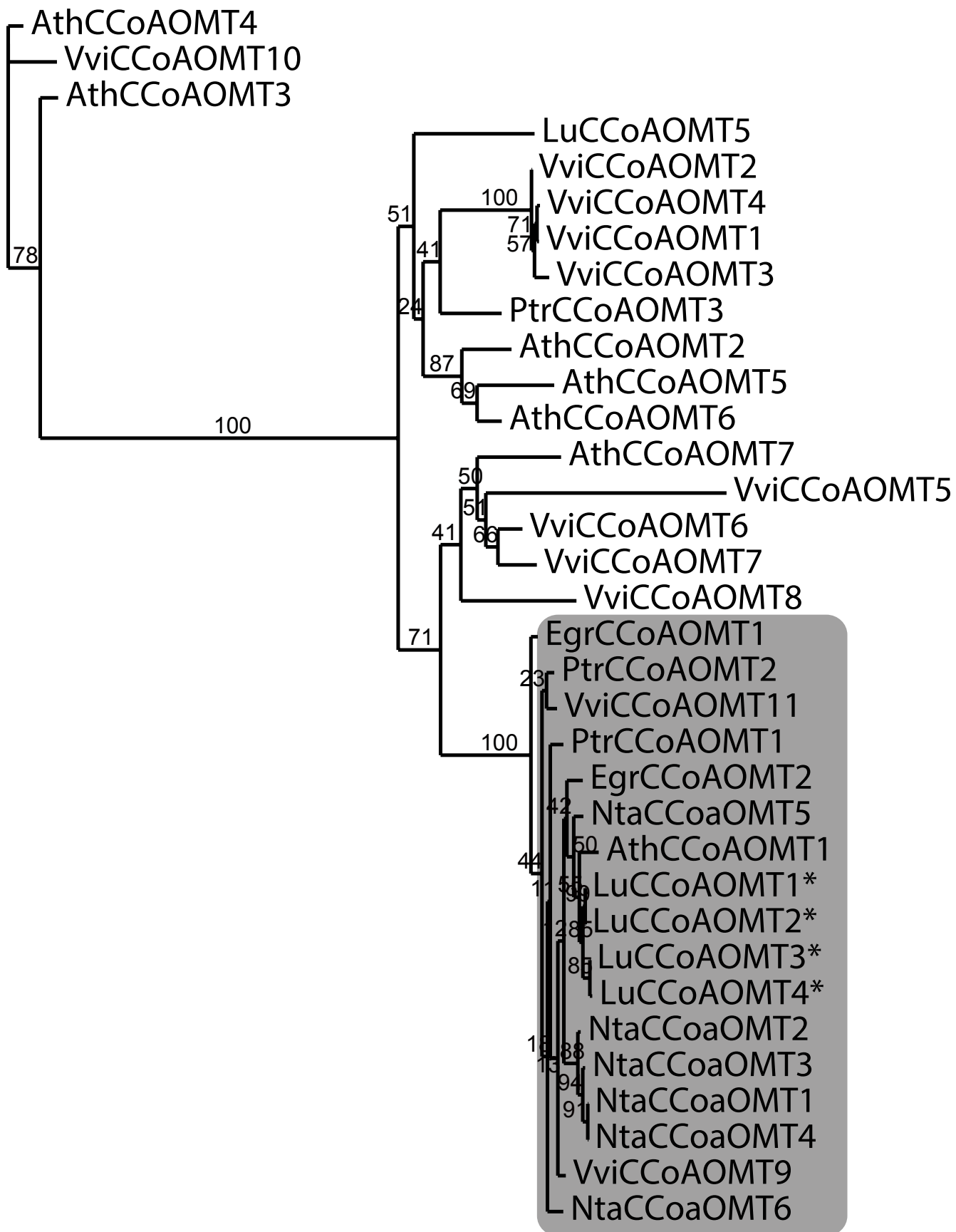

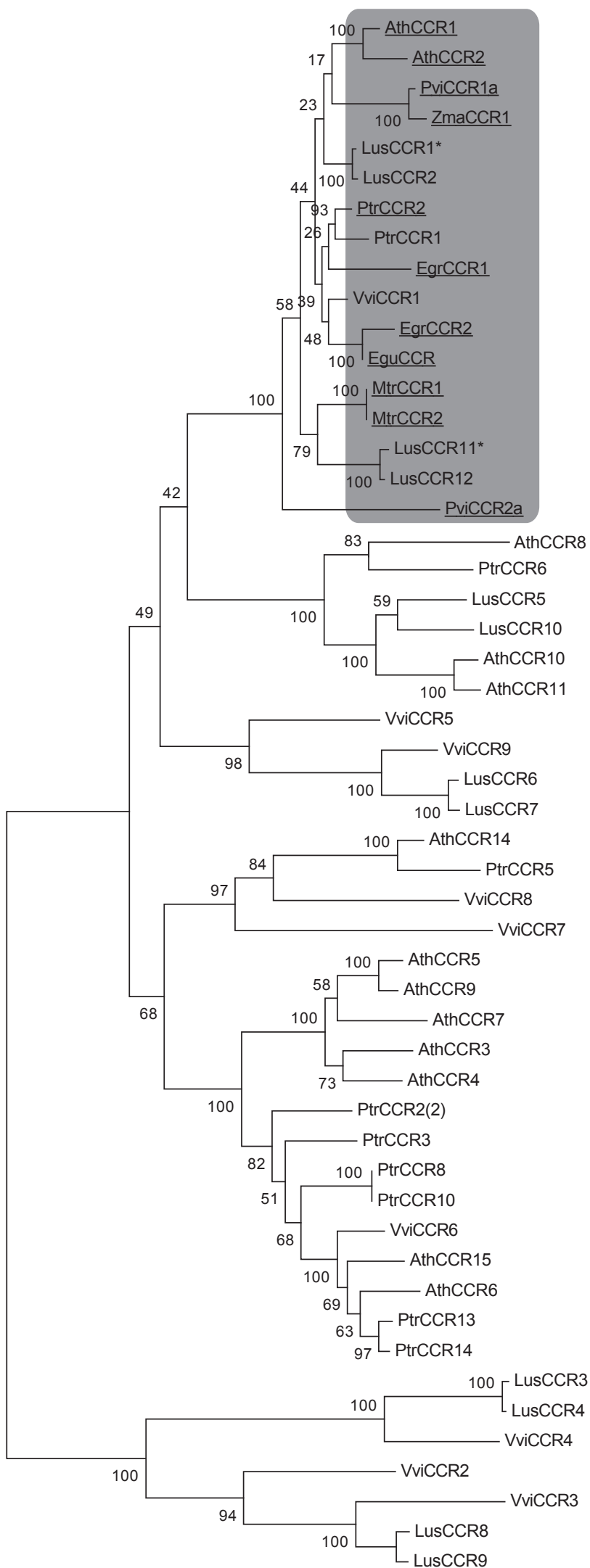

0.2

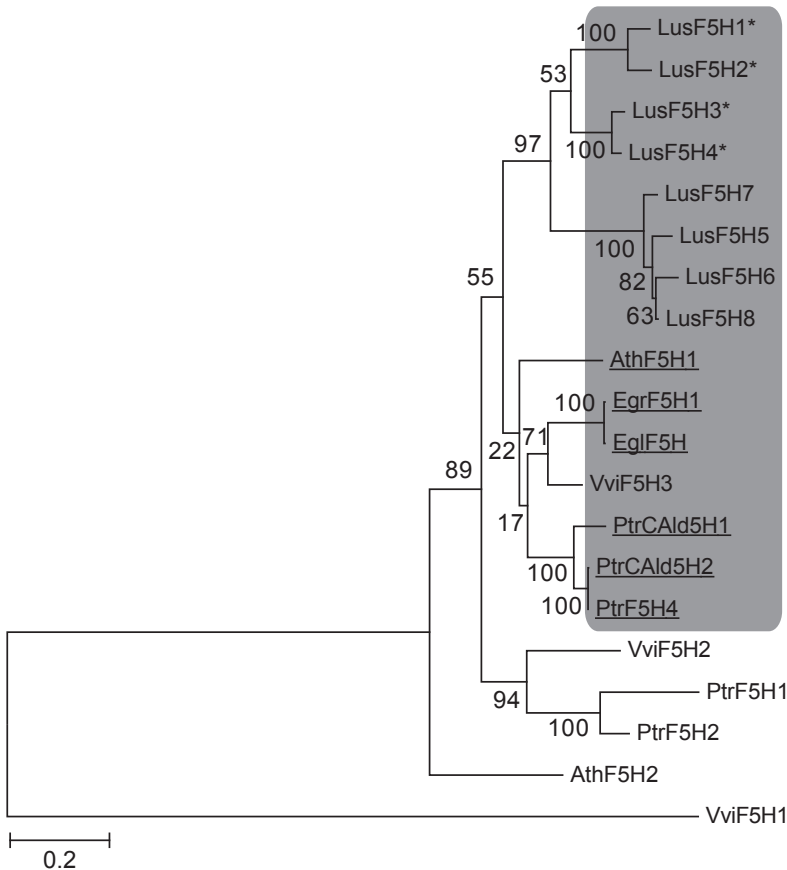

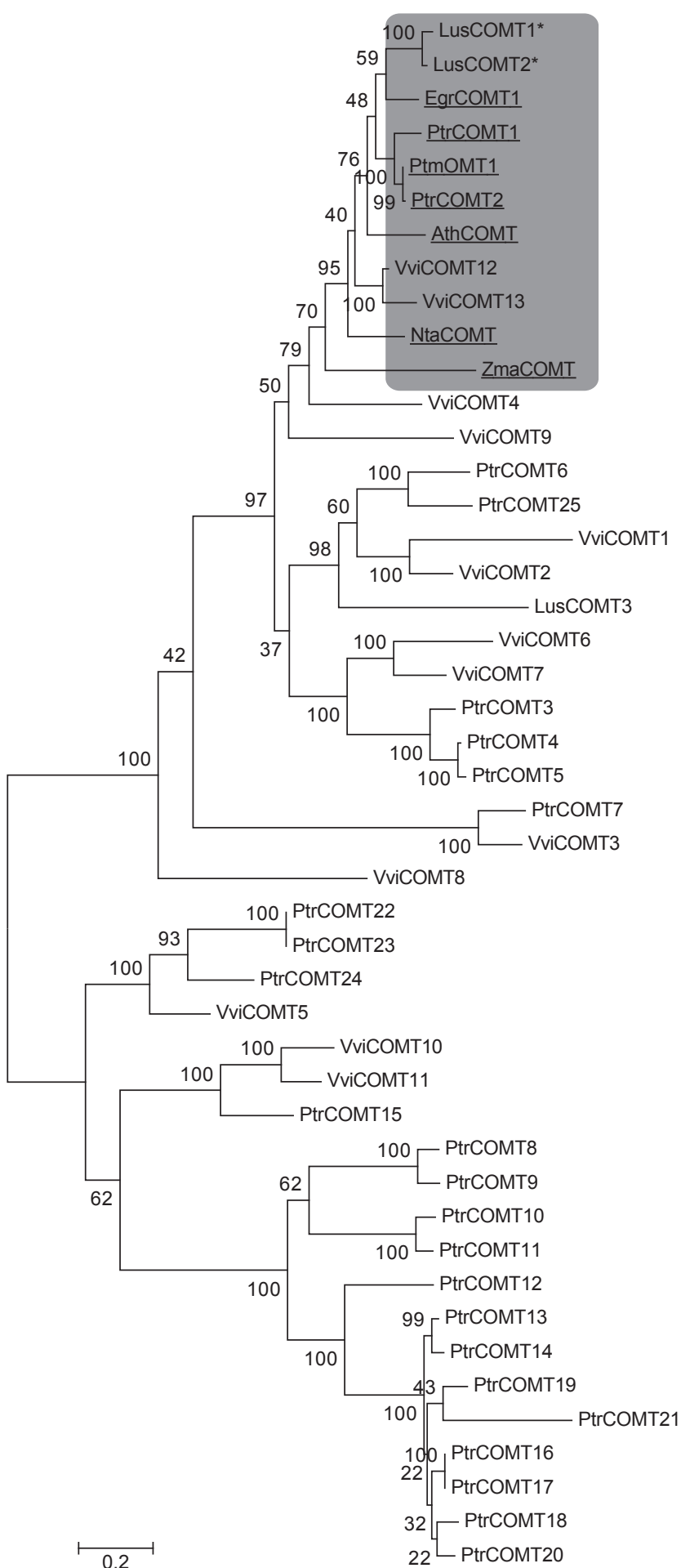

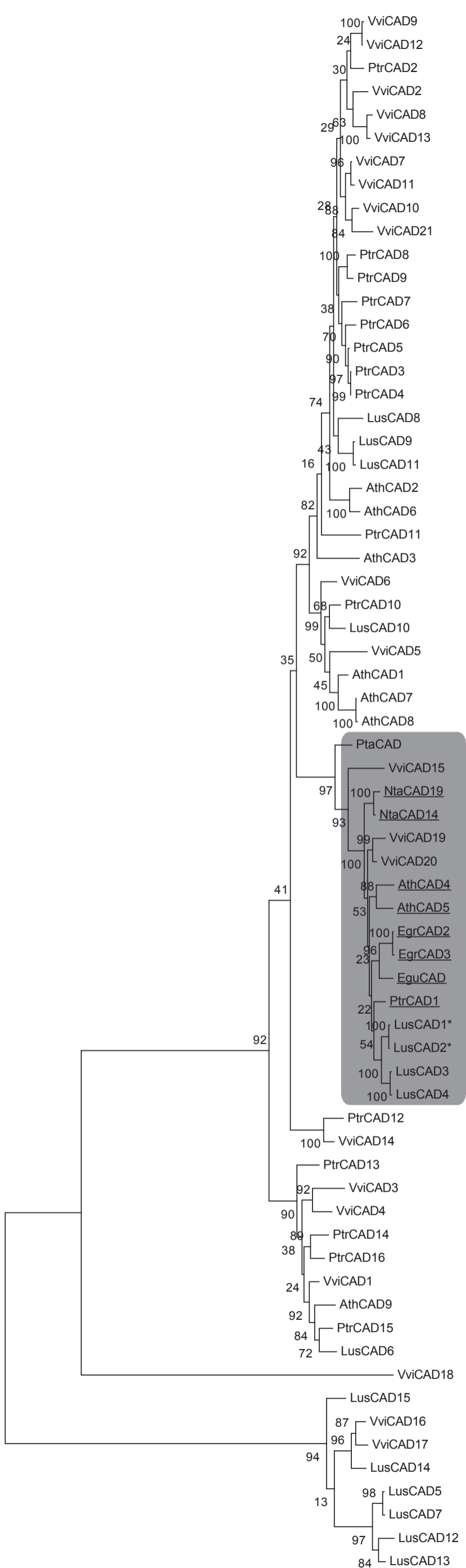

Supplement: Supplementary file 3 — Expression of the flax phenylpropanoid genes under stress conditions as determined by HT-RT-qPCR. 0H: start of the light period on the first day; 48H: end of the night on the second day; 48H_CL: 48 h after continuous light; CONT: control; Dehyd: dehydration; L: leaf; MeJA: methyljasmonate; S: stem; SA: salicylic acid. (PDF 1906 kb) [file 12870_2017_1072_MOESM2_ESM.pdf]

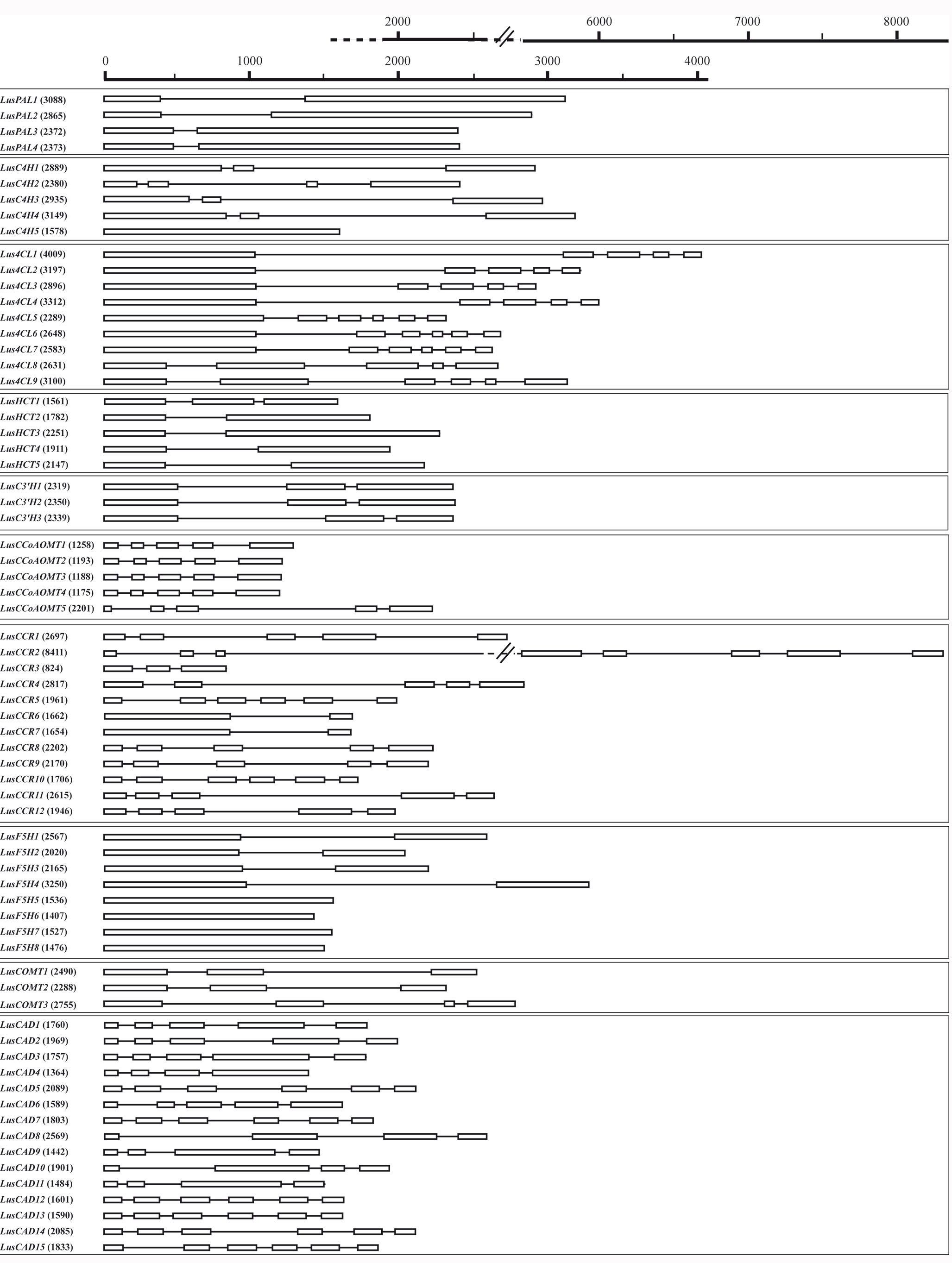

Supplement: Supplementary file 5 — Description of the gene expression localization in the tissues of flax roots, stems and leaves. (JPEG 391 kb) [file 12870_2017_1072_MOESM1_ESM.jpg]
