# Supplementary material for: Spatial regulation of monolignol biosynthesis and laccase genes control developmental and stress-related lignin in flax
Source: BMC Plant Biol. 2017 Jul 14;17:124. doi: 10.1186/s12870-017-1072-9 (PMC5513022; doi:10.1186/s12870-017-1072-9)
Supplement: Supplementary file 4 — Primers used for HT-RT-qPCR, RLM RACE and in situ hybridization. (DOC 76 kb) [file 12870_2017_1072_MOESM6_ESM.doc]

|  | **MBSII** | | **MBSIIG** | |  | **MBSII** | | **MBSIIG** | |
| --- | --- | --- | --- | --- | --- | --- | --- | --- | --- |
|  | + | - | + | - |  | + | - | + | - |
| **LusPAL1** | 119  189 |  | 521  545  605 | 417  1117 | **LusCCR1** |  |  | 1454 | 169 |
| **LusPAL2** | 110  188 |  | 82 | 1422 | **LusCCR2** |  |  | 1028 | 438  601 |
| **LusPAL3** |  |  | 641 |  | **LusCCR3** |  |  | 754  903 | 1496 |
| **LusPAL4** | 871 |  | 944 |  | **LusCCR4** | 1348 |  |  |  |
| **LusC4H1** | 290 |  | 143 | 739 | **LusCCR5** |  |  |  | 134 |
| **LusC4H2** |  |  | 117  174 |  | **LusCCR6** | 816 |  |  |  |
| **LusC4H3** |  |  | 163 | 989 | **LusCCR7** | 216 | 531 | 911 |  |
| **LusC4H4** |  |  | 810 |  | **LusCCR8** | 745 |  | 170  1412 | 1334 |
| **LusC4H5** | 96 | 399 |  |  | **LusCCR9** | 997 | 1031 | 179 |  |
| **Lus4CL1** | 672 |  | 193  217 |  | **LusCCR10** | 797 |  |  |  |
| **Lus4CL2** | 630 | 821 | 190  217 | 757  944 | **LusCCR11** |  |  |  |  |
| **Lus4CL3** |  | 1494 | 244  273  337  511 | 1125 | **LusCCR12** | 1062 | 846 |  |  |
| **Lus4CL4** |  |  | 220  249  370 |  | **LusF5H1** | 1211 | 1295 |  |  |
| **Lus4CL5** |  |  |  |  | **LusF5H2** |  |  |  |  |
| **Lus4CL6** |  | 236 |  |  | **LusF5H3** |  |  | 509 | 1343 |
| **Lus4CL7** |  |  |  |  | **LusF5H4** |  | 1052 |  |  |
| **Lus4CL8** |  |  |  |  | **LusF5H5** | 226 | 532 |  |  |
| **Lus4CL9** |  |  |  |  | **LusF5H6** | 228 | 1119 |  |  |
| **LusHCT1** |  | 903 | 963 | 355  1034 | **LusF5H7** | 231 |  |  |  |
| **LusHCT2** |  | 1081  1142 |  | 340 | **LusF5H8** |  |  | 945 |  |
| **LusHCT3** |  |  |  |  | **LusCAD1** | 1356 |  | 215  1045 |  |
| **LusHCT4** |  |  | 1257 |  | **LusCAD2** |  |  | 211 | 1261 |
| **LusHCT5** |  | 1299  1455 |  |  | **LusCAD3** | 497 |  | 198  1080 |  |
| **LusC3H1** |  | 1268 | 358 |  | **LusCAD4** |  | 132 | 207 |  |
| **LusC3H2** |  | 1263 | 382  429 | 989  1385 | **LusCAD5** |  |  |  |  |
| **LusC3H3** | 105 | 155  868 |  |  | **LusCAD6** |  | 132 |  |  |
| **LusCCoAOMT1** | 178  881 | 1355 | 221  643 |  | **LusCAD7** | 778 |  | 815 |  |
| **LusCCoAOMT2** |  |  | 231  331  709 |  | **LusCAD8** |  |  | 170 |  |
| **LusCCoAOMT3** | 741  850  892 |  | 218 | 708 | **LusCAD9** |  |  |  | 1176 |
| **LusCCoAOMT4** | 566  1084 |  | 205 |  | **LusCAD10** | 681  898 |  |  |  |
| **LusCCoAOMT5** |  |  | 337  615 |  | **LusCAD11** |  |  |  | 1048 |
| **LusCOMT1** |  |  |  | 183 | **LusCAD12** |  | 995 |  |  |
| **LusCOMT2** |  |  |  | 185  740 | **LusCAD13** |  |  |  |  |
| **LusCOMT3** |  | 1145 |  |  | **LusCAD14** |  |  |  | 137  389  526  954  1021 |
|  |  |  |  |  | **LusCAD15** |  |  |  |  |
